# AAV-p40 Bioengineering Platform for Variant Selection Based on Transgene Expression

**DOI:** 10.1089/hum.2021.278

**Published:** 2022-06-10

**Authors:** Adrian Westhaus, Marti Cabanes-Creus, Timo Jonker, Erwan Sallard, Renina Gale Navarro, Erhua Zhu, Grober Baltazar Torres, Scott Lee, Patrick Wilmott, Anai Gonzalez-Cordero, Giorgia Santilli, Adrian J. Thrasher, Ian E. Alexander, Leszek Lisowski

**Affiliations:** ^1^ Translational Vectorology Unit, Children's Medical Research Institute, Faculty of Medicine and Health, The University of Sydney, Westmead, New South Wales, Australia.; ^2^ Great Ormond Street Institute of Child Health, University College London, London, United Kingdom.; ^3^ Biology Department, École Normale Supérieure de Paris, Paris, France.; ^4^ Gene Therapy Research Unit, Children's Medical Research Institute and Sydney Children's Hospitals Network, Faculty of Medicine and Health, The University of Sydney, Westmead, New South Wales, Australia.; ^5^ Stem Cell and Organoid Facility, Children's Medical Research Institute, Faculty of Medicine and Health, The University of Sydney, Westmead, New South Wales, Australia.; ^6^ Stem Cell Medicine Group, Children's Medical Research Institute, Faculty of Medicine and Health, The University of Sydney, Westmead, New South Wales, Australia.; ^7^ Discipline of Child and Adolescent Health, Faculty of Medicine and Health, The University of Sydney, Sydney, New South Wales, Australia.; ^8^ Vector and Genome Engineering Facility, Children's Medical Research Institute, Faculty of Medicine and Health, The University of Sydney, Westmead, New South Wales, Australia.; ^9^ Laboratory of Molecular Oncology and Innovative Therapies, Military Institute of Medicine, Warszawa, Poland.

**Keywords:** AAV, capsid bioengineering, novel vectors, adeno-associated viral vectors, vector development, directed evolution

## Abstract

The power of adeno-associated viral (AAV)-directed evolution for identifying novel vector variants with improved properties is well established, as evidenced by numerous publications reporting novel AAV variants. However, most capsid variants reported to date have been identified using either replication-competent (RC) selection platforms or polymerase chain reaction-based capsid DNA recovery methods, which can bias the selection toward efficient replication or unproductive intracellular trafficking, respectively. A central objective of this study was to validate a functional transduction (FT)-based method for rapid identification of novel AAV variants based on AAV capsid mRNA expression in target cells. We performed a comparison of the FT platform with existing RC strategies. Based on the selection kinetics and function of novel capsids identified in an *in vivo* screen in a xenograft model of human hepatocytes, we identified the mRNA-based FT selection as the most optimal AAV selection method. Lastly, to gain insight into the mRNA-based selection mechanism driven by the native AAV-p40 promoter, we studied its activity in a range of *in vitro* and *in vivo* targets. We found AAV-p40 to be a ubiquitously active promoter that can be modified for cell-type-specific expression by incorporating binding sites for silencing transcription factors, allowing for cell-type-specific library selection.

## INTRODUCTION

Adeno-associated viral (AAV) vectors are well established as gene delivery vehicles in biomedical research, and with three market approvals by the U.S. Food and Drug Administration (FDA) and European Medicines Agency (EMA) to date, their growing clinical dominance is apparent.^1–3^ Numerous naturally occurring and tissue culture “domesticated” variants^4^ enabled early preclinical and clinical development. The last two decades witnessed substantial development in capsid optimization and bioengineering,^5^ with the early fruits of the capsid engineering efforts currently showing great promise in clinical studies.^6^

Despite these early successes, further improvements are required to bring more challenging clinical targets within the technological reach of AAV-based gene therapeutics. Specifically, most AAVs in development today have a limited ability to functionally transduce primary human cells with high efficiency. Novel vectors with increased target specificity would enable therapeutic efficacy at lower vector doses, improving safety, and lowering the cost of AAV-based therapies.^7–9^ To address these rising demands, the global research community has put a significant effort into AAV capsid development, including establishing technologies such as AAV capsid-directed evolution,^10^ randomization strategies for library generation,^11,12^ and sophisticated approaches based on rational AAV capsid modification and design.^13^

Novel vector development through capsid library selection has shown great promise,^5,14–16^ yet the success rate of these selections is not always as high as anticipated. Some published selection platforms allow for linking capsid variant and vector function, either through recombinase enzymes, termed [Multiplexed-] Cre-recombination-based AAV targeted evolution ([M-]CREATE)^17,18^ or iTransduce,^19^ using bicistronic transgenes.^20,21^ Unfortunately, platforms such as CREATE require specific transgenic mice, while the bicistronic designs, which allow enrichment for transduced cells, rely on capsid recovery by polymerase chain reaction (PCR). This makes them susceptible to selecting nonfunctional “passenger” capsids that entered the target cells but do not drive transgene expression.

To reduce the chance of selecting “passenger capsids,” recovery of the capsid gene from expressed RNA would be necessary. More recently, platforms that use ubiquitous or cell-type-restrictive promoters have also been reported (TRACER and DELIVER) to fill that gap.^22,23^ Our own previous studies generated novel approaches to select AAV based on transgene expression driven by the natural AAV *cap* promoter p40.^21,24^ However, early studies have shown that capsid expression from its natural promoter (p40) can be highly inefficient and unreliable,^25^ which poses a challenge considering that most AAV selection platforms are based on the natural AAV2 genome configuration.

In this study, we validated our established AAV selection platform technology that enables the direct use of the native AAV-p40 promoter for capsid recovery from mRNA recovered from functionally transduced target cells.^24^ Furthermore, we developed strategies to enable p40 promoter activity in the range of *in vitro* and *in vivo* targets allowing for universal as well as cell-type-specific AAV selection. In proof-of-concept studies, this new platform has the clear potential to facilitate selection of novel AAVs with an overall higher functionality than AAVs developed with nontransgene expression methods.

## MATERIALS AND METHODS

### Cell culture conditions and cell origins

AAV production was performed using the human embryonic kidney (HEK) 293T cell line (Cat# CRL-3216; ATCC) grown in Dulbecco's modified Eagle's medium (DMEM, Cat# 11965; Gibco) supplemented with 10% fetal bovine serum (FBS, Cat# F9423; Sigma-Aldrich), 1 × Pen Strep (Cat# 15070; Gibco), and 25 mM HEPES (Cat# 15630; Gibco). Human hepatocellular-carcinoma 7 (HuH-7) cells were provided by Dr. Jerome Laurence (The University of Sydney) and cultured in DMEM supplemented with 10% FBS, 1 × Pen Step, and 1 × nonessential amino acids (Cat# 11140; Gibco). Henrietta Lacks (HeLa) cells used in this study were a generous gift from Dr. Hilda Picket (Children's Medical Research Institute [CMRI], Sydney, Australia). The HeLa cells were maintained in DMEM supplemented with 10% FBS and 1 × Pen Strep.

An additional hepatocellular carcinoma cell line (HepG2) and adenocarcinoma cells 549 (A549) were grown under the same conditions as the HuH-7 cells and were provided by Prof. Ian Alexander and Dr. Grant Logan (both CMRI), respectively. Primary human fibroblasts were a gift from Dr. Wendy Gold (Kid's Research, Sydney Children's Hospital Network, Sydney, Australia), and SH-SY5Y cells were obtained from Cell Bank Australia. Both cell types were grown in F12/DMEM (Cat# 11320033; Gibco) with 10% FBS.

Brain organoids were generated as previously described.^26^ Briefly, human induced pluripotent stem cells (iPSCs; UCLOOi017-A-1 and HPSI0314i-hoik_1) were maintained until 90–95% confluent. Media were replaced with Essential 6 media (E6, Cat# A1516401; Life Technologies) for two consecutive days. At day 3 of differentiation, E6 media were replaced with a proneural induction media (composed of advanced DMEM/F12, N2 supplement [Cat# 17502001], l-glutamine [Cat# 25030081], nonessential amino acids [MEM-NEAA; Cat# 11140050], and antibiotic–antimycotic [Cat# 15240096]; all Gibco).

At around week 3–4 of culture, three-dimensional (3D) organoids containing rosettes were observed throughout the plate and in close proximity to neuroretinal vesicles. The 3D cortical organoids were manually isolated and kept together in 60-mm-well plates in retinal differentiation media (composed of DMEM, F12 nutrient mix, B27-vitamin A [Cat# 12587010; Gibco], and antibiotic–antimycotic) and placed on an orbital shaker.

At 6 weeks of differentiation, a retinal differentiation medium was supplemented with 10% FBS, 100 μM taurine (T4871; Sigma), and 2 mM GlutaMAX (Cat# 35050061; Gibco). At 10 weeks, cortical organoids were cultured in a cerebral organoid differentiation medium (CODM; Neurobasal medium, DMEM/F12, N2 supplement, insulin, GlutaMAX, MEM-NEAA, β-mercaptoethanol, B-27 supplement). Differentiation cultures were fed every other day.

### Polymerase chain reactions

Standard and Illumina amplicon-seq PCRs were performed using Q5 (Cat# M0491; NEB), dNTPs (Cat# N0447; NEB), and primers (all Sigma-Aldrich, Supplementary Table S3) as described below using the following standard protocol: initial denaturation: 98°C for 10 s, denaturation: 98°C for 10 s, annealing: unique for each oligo based on https://tmcalculator.neb.com for 10 s, extension: 72°C for 20 s per 1,000 nt, and final extension: 72°C for 10 min. The PCR products were run on agarose gels (Cat# BIO-41025; Bioline), using 1% agarose/1 × Tris-acetate-EDTA (TAE, Cat# 24710-030; Invitrogen) for products over 500 bp, and 2% agarose/TAE for products smaller than 500 bp. The DNA was extracted using the Zymoclean Gel DNA Recovery Kit (Cat# D4001; Zymogen), following the manufacturer's instructions.

### Library preparation

For the AAV2 peptide display library preparation, double *Sfi*I restriction sites were inserted into the local codon-optimized version of the AAV2 *cap* gene (Caplco2) at the N587 insertion site, as described previously (Supplementary Fig. S4).^27^ In brief, using primers with long overhangs and 18 nt homology arms at the 3′end between each other (lco2_*Sfi*I_1 and lco2_*Sfi*I_2, Supplementary Table S3), the pRep2Caplco2 plasmid was amplified and the added regions (primer overhangs bearing *Sfi*I sites) were incorporated using NEBuilder (Cat# E2621; NEB).

The resulting plasmid pRep2Caplco2_*Sfi*I was then digested using *Swa*I and *Nsi*I, and the capsid-containing fragment was ligated into the equally *Swa*I/*Nsi*I digested functional transduction (FT)-spleen focus-forming virus (SFFV) selection platform. This FT-SFFV-lco2_*Sfi*I construct was subsequently digested with *Sfi*I for 18 h at 50°C, purified using the QIAquick PCR Purification Kit (Cat# 28104; Qiagen), and redigested with *Sfi*I for 18 h. Lastly, the construct was dephosphorylated using calf intestinal alkaline phosphatase (Cat# M0290; NEB), using the manufacturer's protocol. The ready-to-use peptide-recipient backbone was run on agarose gel and purified using a gel extraction kit (Cat# D4001; Zymogen).

The peptide library itself was ordered as an oligonucleotide with 20 nt homologies to each end of the *Sfi*I-digested FT-SFFV-lco2_*Sfi*I backbone flanking an NNK_7_ motif coding for randomized amino acids with lower redundancy.^28^ In addition to the seven random amino acids, the “*Sfi*I-clipped” codons upstream (arginine or serine) and downstream (glutamine, lysine, or glutamate) of the random insertion were coded to be semivariable (full oligo as ordered in reverse complement, lco2_NNK7, Supplementary Table S3).

Therefore, the library contained a 7-mer random insert flanked by two variable amino acids coding for 9-mer novel peptides. Before insertion into the backbone, the oligonucleotide was made double-stranded using a short primer binding on the homology arm upstream of the peptide (lco2-dsSyn, Supplementary Table S3), Klenow (exo-) (Cat# M0212; NEB), and dNTPs (Cat# N0447; NEB). The fragment (dslco2-library) was then gel purified and ready for insertion.

The final library was generated by mixing 225 fmol of the digested FT-SFFV-lco2_*Sfi*I backbone with 2,250 fmol of the dslco2-library insert into 13 individual NEBuilder (Cat# E2621; NEB) reactions. The reactions were combined after assembly and purified using ethanol precipitation. The resulting pellet (1 μg of DNA) was used for electroporation into SS320 competent cells (Cat# 60512; Lucigen). The recovered transformants were used to inoculate 250 mL of lysogeny broth (LB) containing 10 μg/mL trimethoprim (TMP). Only 10 μL of recovered transformants were used to plate a 10^−1^ to 10^−5^ dilution series on TMP-LB-agar plates to determine transformation efficiency (2.3 × 10^7^ colonies/μg of DNA). The 250 mL of inoculated LB was maxi prepped (Cat# A31217; Invitrogen).

To move the same library into the replication-competent (RC) platform, the pFT-SFFV-lco2_7mer was digested alongside the pRC platform using *Swa*I/*Nsi*I. One microgram of the RC backbone was ligated with the digested lco2_7mer capsid at a 1:3 ratio and electroporated as described above.

### AAV production

All production types required pAd5 helper plasmids and, in some cases, other helpers, as indicated throughout the section. For studies that aimed to establish the transduction efficiencies in HuH-7 cells, AAV2, AAV8, and AAV-DJ were used for packaging two unique single-stranded *ITR2-CMV-eGFP-N_6_Barcode(BC)-WPRE-ITR2* transgenes each, as described previously.^29^ The 20 novel AAV2 peptide display variants, as well as benchmarks AAV2 and AAV-NP59—for validating novel capsids in primary human hepatocytes *in vivo*—were used for packaging three unique single-stranded *ITR2-LSP-eGFP-N_6_Barcode(BC)-WPRE-ITR2* transgenes each as described previously.^4,21^ The RC-lco2_7mer and FT-SFFV-lco2_7mer libraries (alongside pRep2 helper plasmids) were produced in five 15 cm dishes of HEK293T cells.

For the cytomegalovirus (CMV)/liver-specific promoter (LSP)-green fluorescent protein (GFP) construct, 5 μg of transgene plasmid was transfected per 15 cm dish, while to reduce cross-packaging, ∼100 ng (∼500 copies/cell) of transgene plasmid was transfected per 15 cm dish for library production. All aforementioned constructs were harvested and purified using iodixanol ultracentrifugation, as described previously.^29^

Iodixanol-purified AAVs were quantified using droplet digital PCR (ddPCR; Bio-Rad, Berkeley) using QX200 ddPCR EvaGreen Supermix (Cat# 1864034; Bio-Rad) with eGFP primers, except for the RC-lco2_7mer libraries, which were quantified using Rep2 primers (all primer sequences see Supplementary Table S3), as described previously.^11^

The AAV2, AAV8, and AAV-DJ capsids in all four selection platforms were produced as a single 15-cm-dish crude lysate (*n* = 9) using helpers as indicated in Supplementary Fig. S1a. The same AAVs in the FT and high targeted expression (HTE) platforms were also produced with pRep2-ACG as single 15-cm-dish crude lysates (*n* = 3). AAV-DJ in the FT-LSP/hSyn/MLC platforms was produced using the pRep2 helper as a single 15-cm-dish crude lysate (*n* = 3).

The cross-packaging experiments using AAV2/AAV2 Y576* were performed in single 15-cm-dish polyethylenimine-transfections using indicated amounts of library platform plasmid (*n* = 3). The scAAV-CAG/ITR/p40/SFFVrv-p40/SFFVrv-GFP were also produced as single 15-cm-dish crude lysates (*n* = 1) packaged into the AAV-7m8 capsid.^16^ Crude lysate preparations were performed as previously described.^11^

The resulting AAVs were titrated using real-time quantitative polymerase chain reaction (qPCR) master mix (Cat# 172-5125; Bio-Rad) with serial dilutions of a linearized plasmid as a standard curve and eGFP_F/R and Rep2_F/R primers (Supplementary Table S3).^11^ In addition to benzonase treatment as part of the crude lysate preparation, the clarified lysates were treated with deoxyribonuclease I (DNaseI) (Cat# M0303; NEB) to improve removal of any remaining plasmid or genomic DNA before Proteinase K (Cat# P8107; NEB) digest and subsequent qPCR.

### *In vitro* transductions, flow cytometry

To perform p40 activity studies using scAAVs packaged in AAV-7m8 (see above, section AAV production), cells were seeded at a density of 100,000 cells per well into a 24-well plate using the culture conditions indicated above. The cells were transduced 16 h later at a multiplicity of transduction (MOT) of 1,000 vector genomes (vg) per cell. Subsequently, 48 h after exposure to the vector, the cells were rinsed once with 1 × Dulbecco's phosphate-buffered saline (DPBS, Cat# 14190144; Gibco), dissociated using TrypLE Express (Cat# 12604021; Gibco), and recovered in their respective culture media (see above, section cell culture conditions and cell origins).

Following transfer into 5 mL polystyrene tubes (Cat# 352235; Falcon), the cells were spun down (300 *g*, 10 min, 4°C) and resuspended in FACS+DAPI buffer (DPBS, 2% FBS, 5 mM EDTA [Cat# 15575-020; Invitrogen], 4′,6-diamidino-2-phenylindole dihydrochloride [DAPI, Cat# D9542; Sigma-Aldrich]) to allow the exclusion of dead cells. The flow cytometry analysis was performed using a Becton Dickinson (BD) LSRFortessa cell analyzer at the Flow Cytometry Facility, Westmead Institute for Medical Research (Westmead, NSW, Australia). The data were analyzed using FlowJo 7.6.1 (FlowJo, LLC).

### AAV transduction of brain organoids

AAV vectors (1 × 10^11^ vg/organoid) were added to a total volume of 375 μL of fresh CODM used to culture the cortical brain organoids. The organoids were then transferred to low binding 24-well plates (Costar, Cat# 3524; Corning), and the media were completely replaced with CODM containing the AAV vectors. Cortical and whole-brain organoids were incubated at 37°C for half a day before adding another 625 μL of fresh media. After overnight culture at 37°C, the organoids and CODM/vector mixture were transferred to a 60 mm dish. The dish was topped up with 4 mL with fresh CODM and put on an orbital shaker at 85 rpm at 37°C. After 48 h, organoids were fed every other day.

### Animal work

Mouse studies were supported by the BioResources Core Facility at the CMRI. All animal care and experimental procedures were approved by the joint CMRI and The Children's Hospital at Westmead Animal Care and Ethics Committee. The *Fah*^−/−^*Rag2*^−/−^*Il2rg*^−/−^ (FRG) mice were housed in a BSL2 facility in individually ventilated cages with 10% 2-(2-nitro-4-trifluoro-methylbenzoyl)-1,3-cyclohexanedione (NTBC) supplemented in drinking water.^30^ Six- to 8-week-old FRG females were engrafted with primary human hepatocytes (Lonza Group Ltd., Basel, Switzerland), followed by on/off NTBC cycles to promote the expansion of human cells, as previously described.^4,30–32^

Levels of human hepatocyte engraftment in the chimeric liver were assessed by measuring human albumin levels in peripheral blood using the Human Albumin ELISA Quantitation Kit (Cat# E80-129; Bethyl), as previously described.^30^ Mice were euthanized by isoflurane inhalation 1 week after injection of the vectors for organ and hepatocyte harvests. For fluorescence-activated cell sorting (FACS), hepatocytes were harvested by collagenase perfusion as previously described.^31^

Single-cell suspensions were obtained by cannulation of the inferior vena cava, which was then pumped with an osmotic minipump (Gilson Minipuls 3) in the following order: 25 mL of Hank's Balanced Salt Solution (HBSS) (Cat# H9394; Sigma), 25 mL of HBSS supplemented with 0.5 mM EDTA, 25 mL of HBSS, and 25 mL of HBSS supplemented with 5 mM CaCl_2_, 0.05% w/v collagenase IV, and 0.01% w/v DNaseI.

Following perfusion, the chimeric liver was harvested and placed in a sterile cell culture dish containing DMEM supplemented with 10% FBS. The cells were collected after opening the liver capsule followed by centrifugation at 50 *g* for 3 min at 4°C. The cell pellet was resuspended in cold DMEM and passed through a 100 μm nylon cell strainer. Isotonic Percoll (10% 10 × PBS and 90% Percoll, Cat# 17089102; GE Healthcare) was added to the cell suspension to separate the live and dead cells. Live cells were pelleted at 650 *g* for 10 min at 4°C, and the pellet was resuspended in FACS+DAPI buffer.

To distinguish between murine and human hepatocytes, cells were labeled with phycoerythrin-conjugated anti-human-HLA-ABC (clone W6/32, 1:20, Cat# 12-9983-42; Invitrogen), biotin-conjugated anti-mouse-H2Kb (clone AF6-88.5, 1:100, Cat# 553568; BD Pharmingen), and allophycocyanin-conjugated streptavidin (1:500, Cat# 17-4317-82; eBioscience). Labeled samples were sorted to a minimal 95% purity using a BD AriaIII cell sorter. FACS was performed at the Flow Cytometry Facility, Westmead Institute for Medical Research. The data were analyzed using FlowJo 7.6.1 (FlowJo, LLC).

### AAV2 peptide display library selections

The *in vivo* RC-lco2_7mer library selection in the presence of adenovirus 5 (Ad5) in humanized FRG mice was performed as described previously using the same, previously validated, stock of Ad5.^4^ In brief, 2 × 10^11^ vg of the original RC-lco2_7mer library was injected intravenously into a humanized FRG mouse for the first round of selection, and 250 μL of liver lysate (corresponding to 2 × 10^9^ vg for secondary RC library) was injected intraperitoneally (intravenous injection of clarified liver lysate is highly toxic) for the second round. Before injection and after each of the two rounds of selection, libraries/samples were analyzed by next-generation sequencing (NGS) to track the selection kinetics based on the decreasing number of unique peptides with every round of selection (see below, section Directed evolution using RC and FT platforms in a murine xenograft model of the human liver).

For the *in vivo* FT-SFFV-lco2_7mer library selection, 2 × 10^11^ vg was injected into a humanized FRG mouse. One week after injection, the chimeric liver was perfused, and human HLA- and GFP-positive cells were sorted. DNA and RNA were extracted and RNA was used for cDNA synthesis. DNA and cDNA alongside the original packaged library were used as templates for NGS PCR (see below, section Next-generation sequencing). Subsequently, 2,250 fmol of the peptide library-bearing fragment was inserted into 225 fmol of the twice *Sfi*I-digested and dephosphorylated FT-SFFV-lco2_*Sfi*I using NEBuilder assembly and electroporated into bacteria, as described in the Library preparation section above.

This secondary library bearing the peptides from expressed capsid genes from round 1 was packaged and injected into other humanized FRG mice (4 × 10^10^ vg). The perfusion was performed after 1 week, as explained above, and after the second round, RNA was extracted from human hepatocytes, used for cDNA synthesis, and analyzed by NGS to track selection kinetics.

### DNA and RNA extraction from cells and tissue samples

DNA and RNA were isolated from the cell pellets from the *in vitro* experiments using the AllPrep DNA/RNA Mini Kit (Cat# 80204; Qiagen) following the manufacturer's instructions.

DNA from homogenized mouse tissues and human hepatocytes from humanized FRG mice were isolated using phenol–chloroform extraction after Proteinase K digestion. Briefly, the cells were resuspended in 400 μL of lysis buffer (10 mM Tris-HCl pH 8 [Cat# 15575-020; Invitrogen], 0.1 mM EDTA [Cat# 15575-020; Invitrogen], 0.2% (w/v) sodium dodecyl sulfate [Cat# 71736-100ML; Sigma-Aldrich], and RNase A [Cat# 12091021; Invitrogen]) and incubated at 37°C for 1 h. Afterward, 50 μg/mL of Proteinase K (Cat# 19131; Qiagen) was added and incubated overnight at 55°C at 800 rpm rotation in a heat block (Thermomix, Eppendorf).

The next day, 400 μL of phenol:chloroform:isoamyl alcohol (25:24:1, Cat# P3803-100ML; Sigma-Aldrich) was added and mixed well with the sample. The phases were separated by centrifugation at 21,000 *g* for 15 min and the top aqueous phase containing DNA was transferred to a new tube, mixed with 0.1 × v/v 3 M sodium acetate and 2.5 × v/v 100% ice-cold ethanol, incubated for 1 h at −80°C, and spun at 21,000 *g*, 4°C for 20 min. The resulting DNA pellet was washed twice with 75% (v/v) ethanol and resuspended in nuclease-free water.

RNA from the mouse and human hepatocytes from humanized FRG mice was isolated using 1 mL TriReagent (Cat# T9424; Sigma). In brief, after cell lysis, 200 μL of chloroform (Cat# 22711.26; VWR) was added, mixed, and incubated for 5 min at room temperature and separated using centrifugation at 21,000 *g*, at 4°C for 20 min. Five hundred microliters of aqueous phase was subsequently collected and mixed with 500 μL of cold isopropanol (Cat# 8187661000; Merck EMD). The RNA was pelleted at 21,000 *g*, at 4°C for 20 min. Finally, the RNA pellet was washed twice using ice-cold 75% ethanol and resuspended in nuclease-free water.

### Reverse transcription of extracted RNA

Seven hundred fifty nanograms of total RNA was incubated with two units of TURBO DNase (Cat# AM1907; Invitrogen) twice for 1 h, followed by incubation with the DNase inactivation reagent following the manufacturer's instructions. The DNase-treated RNA was then used for cDNA synthesis using the SuperScript IV First-Strand Synthesis System (Cat# 18091050; Invitrogen) following the manufacturer's instructions using various primers for second-strand synthesis:

- OligodT primer included in the kit for reverse transcription-qPCR experiments evaluating p40 activity with and without helper plasmids.
- A mix of primers adding up to 2 μM consisting of wtAAV8/lco2_R binding wtAAV8 capsid and wtAAV2/DJ_R binding wtAAV2 and DJ capsids used in the 2–8-DJ minilibrary selection.
- Two micromolars of local codon-optimized AAV capsid 2 reverse primer (wtAAV8/lco2_R) for peptide coding region recovery in the FT-RNA library selection in humanized FRG mice.
- Two micromolars of WPRE-binding primer (WPRE_R) to specifically synthesize the barcoded ssAAV-LSP/CMV-GFP-BC-WPRE cDNA used to establish the performance of AAV2, AAV8, and AAV-DJ in HuH-7 cells (CMV) and validate novel capsids in humanized FRG mice (LSP).

### Next-generation sequencing

Cross-packaging experiment: after every production of the cross-packaging experiment, the DNaseI/Proteinase K-treated lysates used for qPCR were also amplified for NGS alongside each Cap2/Cap2_Y576* plasmid mix (for normalization) using the CrossPack_F primer as well as 1 of the 10 barcoded CrossPack_R primers (Supplementary Table S3) for each sample to mix 10 samples into a single NGS reaction (defined as a minimum 1 gigabase yield, often between 2 and 5 gigabases). To establish the amount of cross-packaging, the wt*cap2* and *cap2*_Y576* (T1728G, VP1 numbering) NGS contributions were normalized by multiplication with their specific normalization coefficient (see below, section Normalization of barcoded NGS reads) based on the results of the plasmid mix NGS.

For the minilibrary selection (2–8-DJ), samples were amplified using the 2–8-DJ_F primer and one of six barcoded 2–8-DJ_R primers (Supplementary Table S3) for each sample to allow mixing six samples into a single NGS reaction. The resulting AAV2/8/DJ contributions were normalized to the AAV2/8/DJ input from each experiment. Input templates were the following:

- DNaseI/Proteinase K-treated preselection mixes of 2/8/DJ in the respective platforms (used for normalization).
- RC-Ad5: DNaseI/Proteinase K-treated cell lysate.
- DNA samples: 50 ng of extracted purified DNA from the RC, FT, and HTE platforms.
- RNA: 5 μL of cDNA from FT and HTE platforms.

The AAV2-based peptide display libraries: libraries were analyzed using NGS at every step of selection, including before selection (packaged library), after round 1 (RC-Ad5, FT-DNA, FT-RNA), and after round 2 (RC-Ad5, FT-RNA), using primers PepLib_F/R.

Barcode studies: the AAV2/8/DJ preparations of CMV-GFP-BC-WPRE^29^ (for establishing transduction in HuH-7 cells), the novel AAV2 peptide candidates, and the AAV2 and NP59 controls packaging the LSP-GFP-BC-WPRE^4,21^ (for validating the novel candidates in human hepatocytes *in vivo*) were amplified using 1 of 21 GFP_BC_WPRE_F forward primers and the reverse primer GFP_BC_WPRE_R per sample (Supplementary Table S3). The barcoded scAAV constructs from the p40 promoter study were amplified using the same barcoded forward primers and the universal SV40pA_R (Supplementary Table S3). Input templates were the following:

- The respective mixes of the vectors (used for normalization).
- Fifty nanograms of extracted DNA from cells, mouse tissues, and human hepatocytes.
- Five microliters of cDNA from cells, mouse tissues, and human hepatocytes.

### Normalization of barcoded NGS reads

NGS data obtained from all barcoded samples (excluding the peptide library screen) were normalized to the barcode contribution of the respective preinjection/transduction vector mixes. Read counts for each sample and each variant were multiplied by the variant-specific “normalization coefficient” of the respective input, which was calculated as follows:

$$Normalizationcoefficient=\frac{\%ofNGSreads^{expected}}{\%ofNGSreads^{measured}}$$

The value “% of NGS reads^expected^” depends on the number of barcodes available in the sample. For example, for studies shown in Supplementary Fig. S3a, three vectors (AAV2, AAV8, and AAV-DJ) with two barcodes per vector, for a total of six barcodes in the mix, were used. As an equimolar distribution was to be achieved, each barcode would be expected to account for 16.67% of reads. However, for illustrative purposes, if a given barcode represented 19% of reads (% of NGS reads^measured^), the barcode would be slightly overrepresented and the reads would be multiplied by a normalization coefficient of 0.877 ( = 16.67%/19%).

### Selection of novel capsids

Highly enriched peptide sequences were recovered from NGS reads for all samples. The quality of the NGS results was confirmed by analysis of the results for the constant regions of the NGS amplicon flanking the peptide sequence, reducing the chance that low-quality reads contaminate the analysis. The novel capsids selected were named according to the selection platform (RC or FT) as well as the rank in the library contribution after the second round of selection (Supplementary Table S1). For example, the most highly selected capsid of the RC selection is called RC01.

### Immunofluorescence analysis

Chimeric mouse livers (Fig. 3b–g) were harvested and prepared for imaging 2 weeks after injection following previously published methods.^4^ In brief, livers were fixed with 4% (w/v) paraformaldehyde before being cryoprotected in 10–30% (w/v) sucrose. Livers were then frozen in O.C.T. (Tissue-Tek; Sakura Finetek USA, Torrance, CA). Frozen liver sections (5 μm) were permeabilized in −20°C methanol and then in room temperature 0.1% Triton X-100. Sections were then stained with DAPI (D1306; Invitrogen) at 0.08 ng/mL and an anti-human GAPDH antibody (Clone AF674, Cat# ab215227; Abcam). Images were captured and analyzed on a Zeiss Axio Imager.M1 using ZEN 2 software.

Cortical brain organoids were washed with PBS, fixed for 40–60 min depending on the size of the organoid in 4% paraformaldehyde, and washed again with PBS before overnight suspension in 20% sucrose. The organoids were then embedded in Tissue Freezing Medium (Leica) and snap frozen in liquid nitrogen.

Cortical brain organoids were cryosectioned at 14 μm thickness, collected on SuperFrost slides (Thermo), and preserved at −20°C. Cryosections were washed with PBS and blocked in 5% serum (goat or donkey) in blocking solution (1% bovine serum albumin in PBS with 0.1% Triton-X) for 2 hs.

Primary antibodies (1:200 antiglial fibrillary acidic protein [GFAP], Merck Millipore, 3277611; 1:200 neuronal nuclei [NeuN] clone A60, Merck Millipore MAB377; 1:550 anti-GFP, Thermo Fisher, A21311) diluted in blocking solution were incubated overnight at 4°C. Sections were washed with PBS three times and incubated with a secondary antibody (Alexa Fluor 488, 546, and 633 secondary antibodies; Invitrogen-Molecular Probes) diluted in blocking solution (1:500) at room temperature. Sections were then washed with PBS and counterstained with DAPI (Sigma-Aldrich). For NeuN staining, antigen retrieval was performed. Briefly, slides were placed in target retrieval solution (S1700; Dako) and heated for 90 s in the microwave before blocking.

## RESULTS

### Selection platform designs and manufacturability analysis

**Figure 1.**
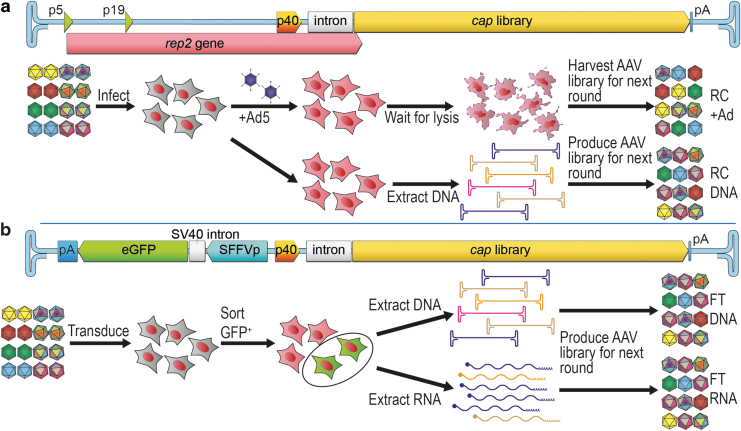
Selection platforms evaluated in this study. **(a)** Conventional RC platform based on the wild-type AAV2 genome, with a capsid library cloned in place of *cap2*. Selection process involves infection of target cells with the RC-library followed by superinfection with wild-type adenovirus and harvest of the cell lysate containing replicated AAV variants and Ad (RC+Ad), which can directly be used for a subsequent round of selection. Alternatively, cells can be infected with the AAV capsid library and DNA can be extracted from infected cells without superinfection-driven AAV amplification, and *cap* genes can be PCR amplified and repackaged for a subsequent round of selection (RC-DNA). **(b)** The FT platform retains the Rep2-p40 promoter to drive the expression of the capsid library. The *rep2* sequences upstream of the p40 promoter are replaced with a reporter cassette, such as an SFFV promoter driving a GFP reporter in the reverse orientation to the p40-*cap*. This configuration forms a bidirectional SFFV/p40-hybrid promoter (SFFVrv-p40). During the selection process, the target cells are transduced with the FT-library, and the subsequently successfully transduced cells are fluorescence-activated cell sorted based on GFP expression. Capsid encoding genes are recovered from DNA (FT-DNA) and/or mRNA (FT-RNA) from the GFP-positive cells, used to produce AAV vectors for a subsequent round of selection or cloning of the final selected AAV candidates. AAV, adeno-associated viral; Ad, adenovirus; FT, functional transduction; GFP, green fluorescent protein; PCR, polymerase chain reaction; RC, replication competent; SFFV, spleen focus-forming virus.

In this study, we utilized three selection platforms, a conventional RC platform (Fig. 1a), an FT platform^21^ (Fig. 1b), and an HTE platform (Supplementary Fig. S1a). The FT platform replaces the AAV2 *rep* gene upstream of the p40 promoter with a reporter cassette expressing a GFP under the control of an SFFV long terminal repeat promoter.^33^ The HTE platform expresses the *cap* ORFs and an internal-ribosomal-entry site (IRES)-linked GFP under the control of an SFFV promoter (see Supplementary Results section “Selection Platform Design” for more detailed descriptions of each platform).

As an initial comparison between the platforms, we investigated their manufacturability and ability to reliably package capsid genes in the corresponding capsid protein. The data strongly suggested that the FT platform packages efficiently irrespective of which promoter was adjacent to p40 (Supplementary Fig. S1) and as reliably as the wild-type RC platform (Supplementary Fig. S2). However, the more synthetic HTE platforms were hampered by their decreased ability to support high-yield packaging (Supplementary Figs. S1 and S2; see Supplementary Results section “Packaging” and “Cross-Packaging” for more detailed results).

### Minilibrary selection on HuH-7 cells indicates a strong selection of the RNA-based FT-directed evolution platform

As an initial validation of the selection platforms, we investigated the stringency and reliability of the selection platforms by performing a mock selection of a minilibrary composed of three AAV variants. To this end, AAV2, AAV8, and AAV-DJ were packaged in all platforms and selected against one another in HuH-7 cells (Supplementary Fig. S3 and Supplementary Results section “Mini Library Selection”).

Based on the known superior performance of AAV-DJ on HuH-7 cells (Supplementary Fig. S3a, RNA expression), it was hypothesized that the stringency of individual platforms could be evaluated based on their ability to select for AAV-DJ. The data showed that the two selection platforms most stringently enriching for AAV-DJ were the RC platform at high multiplicity of infection (MOI) with human Ad5 superinfection and the FT platform when recovering capsids from RNA (Supplementary Fig. 3b).

The FT platform with DNA-based recovery and the RC platform at low MOI also led to AAV-DJ enrichment, although the selection was less efficient. Based on the data from the packaging validation experiments (Supplementary Figs. S1 and S2) and the results of the minilibrary selection (Supplementary Fig. S3b), we concluded that the most reliable and stringent AAV library selection methods were the FT platform with RNA-based recovery and RC-Ad5 platform (Supplementary Fig. S3b).

### Directed evolution using RC and FT platforms in a murine xenograft model of the human liver

While the HuH-7 studies discussed above showed that both FT-RNA and RC-Ad5 selections were equally efficient, we wanted to perform a more stringent comparison in the context of a high complexity library and advanced model system of the human liver. To this end, we compared those selection approaches in the context of AAV-directed evolution selections on primary human hepatocytes in a xenograft model *in vivo* using a highly variable AAV capsid library.

Specifically, we constructed an AAV2 peptide display library (Supplementary Fig. S4) and inserted it into RC and FT library constructs. Both libraries were used to perform AAV selection on human hepatocytes in the xenograft FRG^30^ mouse. This preclinical model of the human liver was chosen because primary human hepatocytes are permissive to Ad5 infection and the use of the RC platform is well established and optimized for this model.^4,15,34^

**Figure 2.**
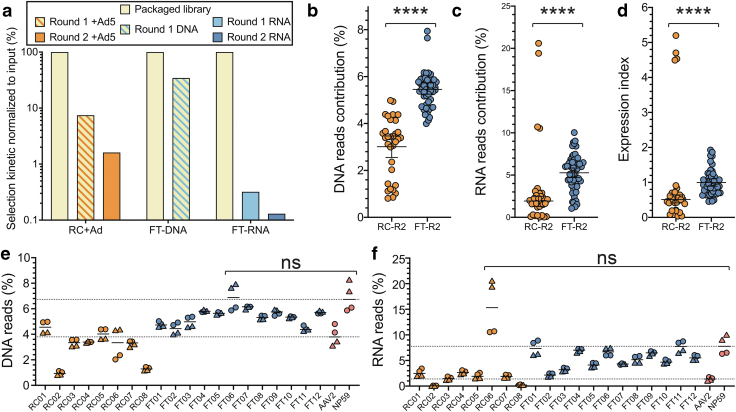
*In vivo* selection of an AAV2-based peptide display library using the RC and FT platforms in human primary hepatocytes *in vivo*. **(a)** Comparison of selection kinetics between the RC and FT platforms. **(b–d)** The performance of the most enriched variants from RC-Ad5 selection (*orange*) and FT-RNA selection (*blue*) at the level of cell entry (DNA) **(b)**, transgene expression (RNA/cDNA) **(c)**, and vector EXI (RNA reads normalized to DNA reads) **(d)**. Statistics: Mann–Whitney ****p-value: <0.0001. **(e, f)** Individual performance of the most abundant variants from RC-Ad5 selection (*orange*) and FT-RNA selection (*dark blue*) **(b, c)** at the DNA (cell entry) **(e)** or the RNA/cDNA (expression) **(f)** levels. AAV2 (*lower dotted line*) and NP59 (*upper dotted line*) were included as controls. Experiments were performed in four humanized mice that had engrafted human hepatocytes from either an adult male (*circles*) or an infant female (*triangles*) donor. Statistics are shown for each variant with a higher average performance compared with NP59. Statistics: Mann–Whitney with Holm–Sidak multiple comparison adjusted *p*-value: ns ≥0.05. EXI, expression index.

When using the RC platform, we observed retention of 7.45% of the candidates present in the original library after the first round and 1.6% after the second round of selection (Fig. 2a and Supplementary Table S1), consistent with a strong selection process. In comparison, after one round of FT-RNA selection, we detected retention of only 0.3% of unique peptides, which was further reduced to 0.1% after the second round of selection (Fig. 2a).

To validate that the more rapid kinetics of selection with the FT platform was driven by the RNA-based recovery, we analyzed FT selection at the DNA level (Fig. 2a). As anticipated from the minilibrary selection on HuH-7 cells (Supplementary Fig. S3b), the FT selection process was less efficient when DNA was used for capsid recovery. Specifically, we detected 34.5% of unique peptide variants from the initial library after one round of FT-DNA selection in GFP-positive sorted human hepatocytes (Fig. 2a, FT-DNA).

To study which of the two selection platforms supported the selection of the most functional AAV vectors, the top 8 candidates from the RC-Ad5 selection (NGS read contribution >1%, Supplementary Table S1) and the top 12 candidates from the FT-RNA selection (NGS read contribution >2.5%, Supplementary Table S1) were vectorized. All novel variants were used for packaging unique barcoded ssAAV.LSP-GFP cassettes.^4,21^

To exclude potential bias caused by the barcode, three uniquely barcoded cassettes were packaged per vector. The prototypical AAV2^4^ and the strong human hepatocyte-tropic AAV-NP59 were included as controls. All 22 vectors were mixed at an equimolar ratio and were injected intravenously (2.00 × 10^11^ total vg/mouse, or 9.09 × 10^9^ vg/variant/mouse) into four humanized FRG mice, of which two were engrafted with hepatocytes from a male and two from a female human donor.

Analysis of the capsids recovered from the RC-Ad5 and FT-RNA selections revealed that, on average, the capsids recovered from the FT-RNA selection performed significantly better at the level of cellular vector genome abundance at the time of harvest (DNA) (Fig. 2b) and expression (RNA/cDNA) (Fig. 2c) in primary human hepatocytes *in vivo* in the humanized FRG mouse.

In addition, analysis of the average expression indices (EXIs) (RNA read contribution divided by DNA contribution)^21^ revealed that once FT-selected capsids enter human hepatocytes, they are significantly more efficient than the RC-selected variants at contributing to RNA expression in these cells (Fig. 2d). Collectively, the results from the selection kinetics (Fig. 2a) as well as the overall functionality (Fig. 2b–d) show that the FT-RNA selection strategy is more stringently enriching for functional novel variants than RC-Ad5.

Interestingly, and in support of the effectiveness of the FT-RNA platform, an analysis of the performance of the individual 20 novel capsids revealed that the top variant selected using the RC-Ad5 strategy (AAV-RC01, 76% of all reads after the second round of selection, Supplementary Table S1) was not a highly functional variant. Based on DNA entry (median: 4.6%) and RNA expression (median: 2.5%), AAV-RC01 was found to be only slightly better than AAV2 (median: 3.8% and 1.4%, respectively, Fig. 2e, f).^4,21,34^

A surprising observation was that another variant, AAV-RC06, which was not efficient at cell entry (DNA, median: 3.3%, Fig. 2e), was highly efficient at driving RNA expression (median: 15.3%), even more efficient than the positive control vector AAV-NP59 (median: 7.8%; Fig. 2f). All capsids recovered from RNA using the FT selection platform were more efficient at cell entry and expression than AAV2 (Fig. 2e). At the transgene expression level (RNA), four candidates (AAV-FT01, AAV-FT04, AAV-FT06, and AAV-FT11) were found to be comparable with highly human hepatocyte-tropic AAV-NP59 (median: 7.8%, Fig. 2f).

**Figure 3.**
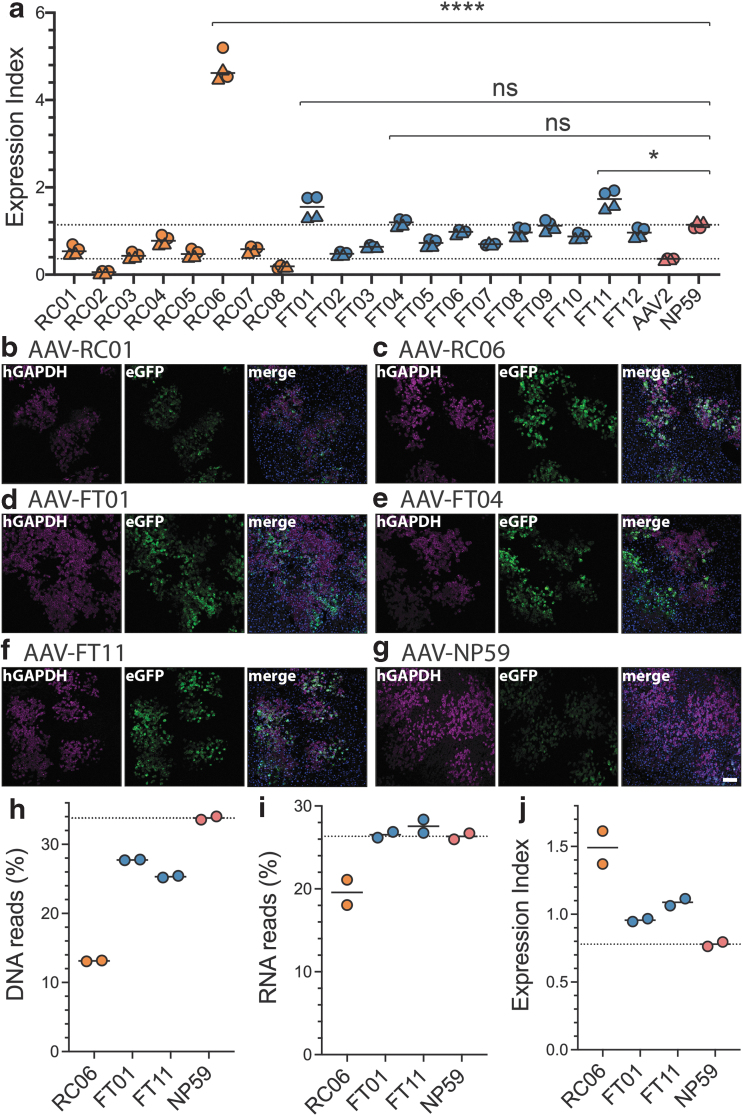
Functional analysis of AAV2-peptide capsid variants in primary human hepatocytes in hFRG mice. **(a)** EXI of individual capsid variants compared with AAV2 (*lower dotted line*) and NP59 (*upper dotted line*) [deconvoluted data from Fig. 2d]. Experiments were performed in four humanized mice that had engrafted human hepatocytes from either an adult male (*circles*) or an infant female (*triangles*). Statistics are shown for each variant with a higher average EXI compared with NP59. Statistics: Mann–Whitney with Holm–Sidak multiple comparison adjusted *p*-value: ns ≥0.05 > * ≥ 0.01/0.0001 > ****. **(b–g)** Representative immunofluorescence images of liver sections from humanized FRG mice transduced with indicated AAV variants. *Blue*: DAPI, *red*: human GAPDH, *green*: native AAV-encoded GFP. The scale bar is 100 μm and valid for all images. **(h–j)** NGS analysis on DNA **(h)**, RNA **(i)**, and EXI **(j)** of indicated capsids obtained from separate transductions at doses of 9.09 × 10^9^ vector genomes/variant/mouse using two xenograft mice with very highly engrafted livers. DAPI, 4′,6-diamidino-2-phenylindole dihydrochloride; GAPDH, glyceraldehyde-3-phosphate dehydrogenase; hFRG, human *Fah*^−*/*−^*/Rag2*^−*/*−^*/Il2rg*^−*/*−^; NGS, next-generation sequencing.

To select variants for further analysis, we evaluated the EXI^21^ of the individual novel and control variants (Fig. 3a). Analysis revealed that three of the four novel FT-selected variants that led to a high expression at the RNA level were also able to achieve higher EXI (medians: AAV-FT01: 1.6; AAV-FT04: 1.2; AAV-FT11: 1.7) than AAV-NP59 (median: 1.1, Fig. 3a), with variant AAV-FT11 showing a significant increase in EXI over AAV-NP59. Not surprisingly, based on data presented in Fig. 2f, AAV-RC06 had the highest EXI of all the capsids tested (median: 4.6, Fig. 3a). This highly functional variant was also represented by the outlier data points observed at the RNA and EXI levels in RC-R2 groups (Fig. 2c, d).

Based on these results, we evaluated the performance of the top 4 variants (AAV-RC06, AAV-FT01, AAV-FT04, and AAV-FT11) in individual humanized FRG mice using immunofluorescence (IF) analysis following tail vein injection of 1 × 10^11^ vg per mouse. We also evaluated AAV-RC01, which despite being the most strongly selected variant in the RC selection showed weak expression based on NGS. We thus hypothesized that IF staining was an excellent tool to validate the NGS results. AAV-NP59 was included as a positive control (Fig. 3b–g and Supplementary Fig. S5).

We found that all novel capsid variants had the same level of specificity to human hepatocytes as AAV-NP59, given that all GFP-positive cells appeared to be positive for the human cell marker glyceraldehyde-3-phosphate dehydrogenase (hGAPDH) (Fig. 3b–g). Moreover, as expected based on lower EXI (Fig. 3a), the variant AAV-RC01 showed the lowest level of GFP expression, which was especially evident when examining the chimeric liver using ultraviolet light before harvest (Supplementary Fig. S5a).

IF staining revealed that AAV-NP59 led to a relatively weak GFP expression compared with the novel AAV variants RC06, FT01, FT04, and FT11, an interesting result given the RNA/cDNA NGS data (Fig. 2f) and the EXI (Fig. 3a). However, the effective vector dose per human cell depends partly on the engraftment level of human hepatocytes in the FRG mouse, the replacement index (RI),^35^ which could affect the strength of the GFP expression.

To address the potential impact of RI on the results of our study, we evaluated this effect using the control AAV-NP59 vector. To this end, we transduced a low engrafted FRG mouse (hAlb level of 0.73 mg/mL, estimated RI <10% human, Supplementary Table S2) with the same vector dose as in the studies discussed above (Fig. 3g).

As hypothesized, this resulted in a substantially stronger GFP expression per human cell (Supplementary Fig. S5g) driven by a higher effective MOT. Based on these results, we performed the analysis of the direct comparisons between the AAV variants in animals with matching engraftment levels (RC01 [0.77 mg/mL hAlb, Fig. 3b] vs. NP59-low [0.73 mg/mL hAlb, Supplementary Fig. S5g] and FT11 [4.4 mg/mL hAlb, Fig. 3f] vs. NP59-mid [4.4 mg/mL hAlb, Fig. 3g]), which revealed that while AAV-RC01 was less efficient than AAV-NP59 at transgene expression in human hepatocytes, AAV-FT11 was substantially more efficient than AAV-NP59. Both of these observations match the EXI quantification analysis (Fig. 3a).

Lastly, to investigate the impact of the RI on NGS analysis, we performed a comparison of the most promising variants (RC06, FT01, and FT11) with AAV-NP59 in two very highly engrafted mice (RI: >90%, Fig. 3h–j). At the DNA level, AAV-NP59 performed more efficiently than the new variants, similarly to what was previously observed in low engrafted mice (Fig. 2e). At the RNA expression level, FT01, FT11, and NP59 performed at similar levels, with NP59 being slightly weaker than the two novel variants (Fig. 3i).

In contrast to what was observed previously, RC06 was less efficient at the transgene level expression (RNA) in highly engrafted mice compared with the low engrafted mice (Figs. 2f vs. 3i). However, in the context of the EXI, we observed the same trend as in low engrafted mice with RC06>FT11>FT01>NP59 (Fig. 3a vs. j). While these data clearly show that capsids are differently affected by varying replacements indices, the observed data for the FT-selected capsids further support the potential selective advantage of the FT-RNA selection method over the conventional RC platform.

### The native AAV p40 promoter is ubiquitously active

**Figure 4.**
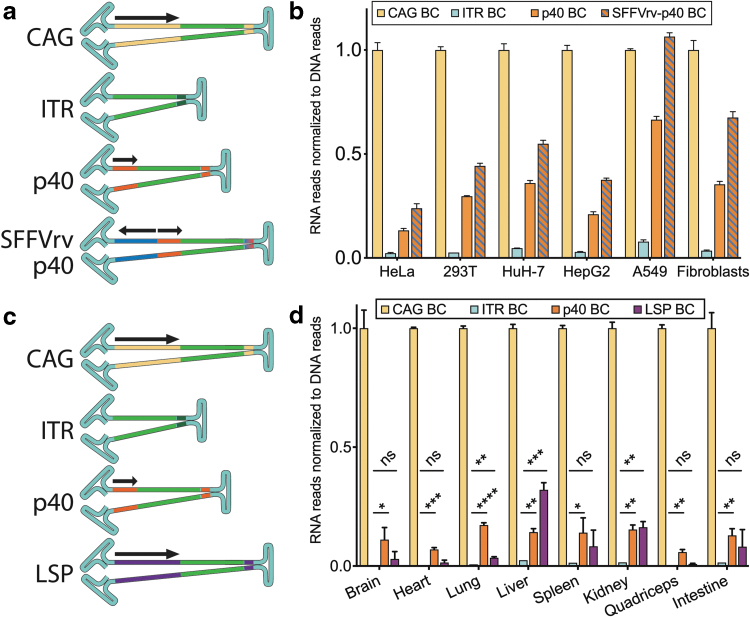
Rep2-p40 activity *in vitro* and *in vivo*. **(a, b**) Uniquely barcoded scAAV constructs used in the study shown in **(b)**. *Arrows* indicate the direction in which the promoters are orientated. EXI based on Illumina sequencing of RNA and DNA purified from cells transduced with AAV-7m8 encoding barcoded scAAV-“Promoter”-GFP constructs from **(a)**. To obtain EXI, the RNA/cDNA signal was adjusted to the entry of each construct (DNA reads). The final EXI was subsequently normalized to scAAV-CAG-GFP, which was assigned a value = 1. MOT = 500 and 5,000 vector genome per cell, *N* = 1 for each MOT for all cell types, values shown are averages of both MOTs. **(c, d**) Uniquely barcoded constructs **(d)** packaged into scAAV9 were used to transduce mouse *in vivo*. Illumina sequencing was used to analyze vector entry (DNA) and expression (RNA) in indicated tissues, and results were used to calculate an EXI. The final EXI was subsequently normalized to scAAV-CAG-GFP, which was assigned a value = 1. Total vector dose = 1 × 10^11^ vector genome copies per mouse, intravenous injection, *N* = 3 for all tissues. Statistics: Nonparametric multiple comparison's *t*-test (Holm–Sidak) compared with ITR indicated as *asterisk* for adjusted *p*-values: 0.05 > * ≥ 0.01 > ** ≥0.001 > *** ≥0.0001 > ****. CAG, cytomegalovirus enhancer–chicken β-actin promoter–globin intron; ITR, inverted terminal repeat; LSP, liver-specific promoter (apolipoprotein E enhancer/human alpha antitrypsin promoter); MOT, multiplicity of transduction; p40, Rep2-p40 promoter.

Having established the strong selective advantage of the FT-RNA selection method, to fully understand the potential impact of this method, we performed further experiments to establish the activity of the native AAV-p40 promoter in multiple cellular targets. Initial experiments demonstrated that the p40 promoter (wtAAV2 genome, NC_001401, 1,700–1,853 nt^25^) had detectable activity without the Rep2 or Ad5 proteins present, especially when linked in reverse to the SFFV promoter (configuration of p40 in the FT platform; Supplementary Fig. S6 and Supplementary Results section “Activity of the Native AAV-p40 Promoter”). This was further elucidated using NGS-based screening with uniquely barcoded self-complementary (scAAV) constructs (Fig. 4a; see Supplementary Results section for more details).

Four constructs were designed to express GFP under the control of (1) a strong ubiquitous CAG promoter (CMV enhancer, chicken beta-actin promoter, and rabbit globin intron), (2) the “promoter-less” background expression from the D-sequence of the AAV2 inverted terminal repeat (ITR; ITR-only),^36,37^ (3) the AAV2-p40 promoter, or (4) the SFFVreverse-p40 dual back-to-back promoter (p40 promoter with an SFFV promoter cloned upstream in reverse orientation (rv) as present in the FT platform, SFFVrv-p40, Fig. 1b). All constructs were packaged into AAV-7m8, shown to be highly efficient at *in vitro* transduction of multiple cell types,^29^ and used to transduce target cells at MOTs of 500 and 5,000 vg per cell.

For cell lines HeLa, HepG2, and A549, the NGS results were in agreement with the GFP mean fluorescence intensity results obtained by flow cytometry (Fig. 4b and Supplementary Fig. S6). The other cell lines (HEK293T and HuH-7) and primary fibroblasts followed the same trend (CAG>SFFVrv-p40>p40>ITR; Fig. 4b). Therefore, we hypothesized that the SFFVrv-p40—and hence the FT platform—could drive *cap* gene expression in a range of target cells *in vitro*.

Having shown the activity of the p40 promoter in cells *in vitro*, we next evaluated the activity of this endogenous AAV promoter *in vivo* in mice to confirm that the FT platform could be universally applicable for AAV selection screens. Uniquely barcoded self-complementary (scAAV) constructs expressing GFP from the strong ubiquitous CAG promoter, the “promoter-less” AAV2 ITR,^36,37^ the AAV2-p40 promoter, or the full-length LSP (apolipoprotein E enhancer,^38^ and human alpha-1 antitrypsin [hAAT] promoter,^39^ 1,066 bp)^21^ were packaged into the AAV9 capsid (Fig. 4c). We injected 1 × 10^11^ vg per mouse into three naive (nonengrafted) FRG mice.

One week after injection, tissues were harvested, processed, and GFP transgene expression was analyzed at the RNA level by NGS. Normalizing expression (RNA) reads to vector cell entry (vector DNA) showed that the CAG promoter was the strongest among the constructs tested (Fig. 4d). Transcripts expressed from the “promoter-less” ITR construct were detected in all tissues, however, the expression was very low (Fig. 4d).

Importantly, transcripts driven by the p40 promoter were readily detectable in all samples (Fig. 4d) and significantly different from the ITR construct, indicating that p40 functions as a ubiquitous promoter in all the tissues analyzed (Fig. 4d). The LSP displayed significant levels of expression only in the liver, lung, and kidney (Fig. 4d) when compared with the “promoter-less” ITR construct (Fig. 4d), which is consistent with our previous observations in mouse kidneys (not shown). While the above background activity of the LSP detected in the lung was unexpected, it can potentially be explained by the low hAAT activity in that organ.^40^

### The AAV p40 promoter's activity can be modified using additional promoter elements

Building on the *in vitro* results which indicated that SFFVrv could increase the expression strengths of the p40 promoter in cell lines and primary fibroblasts (Fig. 4b), additional p40-hybrid promoters were constructed (Supplementary Fig. S7a) to elucidate this phenomenon further. In addition to SFFV, a minimal version of the LSP (456 bp mLSP, as previously described^21^) and the neuron-specific human synapsin 1 (hSYN)^41^ promoter was used to drive the GFP in forward orientation and additionally in a separate construct in back-to-back orientation with p40 driving GFP.

**Figure 5.**
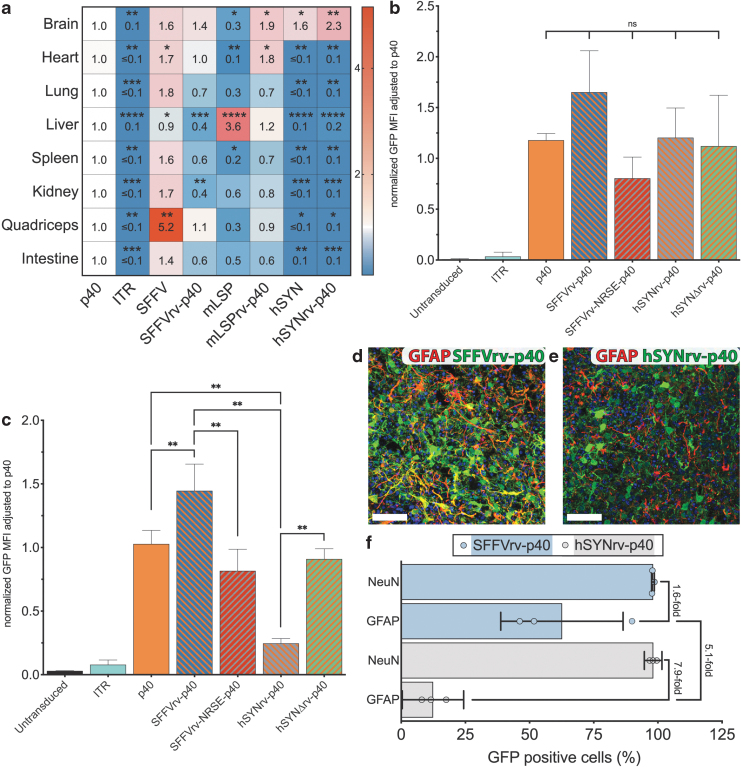
Modification of the Rep2-p40 promoter to enhance cell-type specificity *in vitro* and *in vivo*. **(a)** EXI of barcoded scAAV-“Promoter”-GFP constructs (Supplementary Fig. 7a) adjusted to scAAV-p40-GFP. Equimolar vector mix was injected into *n* = 3 naive mice at a dose of 1 × 10^11^ vector genome copies per mouse. Statistics: Nonparametric multiple comparison's *t*-test (Holm–Sidak) compared with p40 indicated as *asterisk* for adjusted *p*-values: 0.05 > * ≥ 0.01 > ** ≥0.001 > *** ≥0.0001 > ****. **(b, c)** Median (*n* = 6) GFP fluorescence of AAV-7m8 with either AAV-p40 or indicated hybrid promoters (Supplementary Fig. 7b) driving GFP expression in neuroblastoma cells (SH-SY5Y) **(b)** and HeLa cells **(c)** Statistics: Nonparametric *t*-test (Mann–Whitney) compared as indicated, *p*-value: ns ≥0.05/0.01 > ** ≥0.001. **(d, e)** Representative images of iPSC-derived cortical organoid transduced with AAV-7m8.SFFVrv-p40-GFP **(d)** or AAV-7m8.hSYNrv-p40-GFP **(e)**. All cells were stained with DAPI (*blue*), GFAP-positive cells were stained for GFAP expression (*red*), and transduced cells were stained *green* for using the anti-GFP antibody. Scale bar represents 50 μm. **(f)** Quantification of transduced neuronal and GFAP-positive astrocytes in experiments shown in **(d, e)**. Three organoids of different iPSC origins were transduced with each construct and 10 pictures were evaluated for each organoid. Medians of each organoid are shown as *circles* (*light blue* for SFFVrv-p40, and *light gray* for hSynrv-p40). The *horizontal bars* represent the average GFP positivity of neuronal nuclear antigen (NeuN)-positive neurons and GFAP-positive glial cells. *Light blue bars* represent experiments using SFFVrv-p40, and *light gray bars* represent hSYNrv-p40 experiments. GFAP, glial fibrillary acidic protein; HeLa, Henrietta Lacks; hSYN, human synapsin 1 promoter; hSYNΔ, human synapsin I promoter with deleted NRSE; iPSC, induced pluripotent stem cell; mLSP, minimal liver-specific promoter; NRSE, neuron restrictive silencing element.

All constructs were uniquely barcoded, packaged into AAV9, and used *in vivo* followed by NGS expression analysis alongside p40 alone and the no-promoter “ITR-GFP” control as described above. NGS data from cDNA were normalized using the reads from vector DNA, and p40 reads were assigned the value 1 as a reference (Fig. 5a).

In all tissues, expression of GFP reporter was significantly weaker under the control of AAV ITR than the p40 promoter (Fig. 5a). The SFFV promoter drove strong expression of GFP in all tissues, except the murine liver (Fig. 5a). In contrast to experiments *in vitro*, the SFFVrv-p40 promoter did not universally express stronger than p40 and even showed a significantly reduced expression in the liver and kidney (Fig. 5a).

As expected, mLSP drove a very strong expression in the liver (Fig. 5a) and showed reduced expression elsewhere (Fig. 5a). Linking mLSP to p40 in rv resulted in an mLSPrv-p40-driven expression that was significantly increased in the brain and heart compared with p40 alone, but only modestly increased in the liver, indicating that the mLSPrv could not specifically enhance p40 activity in that organ (Fig. 5a). The reportedly neuron-specific hSYN promoter only showed expression in the brain (Fig. 5a) and, importantly, hSYNrv-p40 showed the same expression pattern as the hSYN promoter alone, indicating that the ubiquitous activity of p40 was restricted to the brain by elements in the hSYN promoter (Fig. 5a).

The repressor element 1/neuron restrictive silencing element (RE-1/NRSE),^42,43^ naturally occurring in hSYN, was reported to be responsible for silencing hSYN and hSYNrv-p40 outside of the brain (Supplementary Fig. S7b).^41^ We hypothesized that by placing p40 adjacent to hSYNrv, the activity of p40—and therefore the expression of the FT-hSYN capsid library—could be neuron-specific (Supplementary Fig. S1g, h). To this end, we created two new constructs: one incorporated the RE-1/NRSE site into SFFVrv-p40 (scAAV.SFFVrv-NRSE-p40-GFP), while the other contained hSYNrv-p40 with the deletion of RE-1/NRSE (scAAV.hSYNΔrv-p40; Supplementary Fig. S7b).

The constructs were packaged in AAV-7m8 and tested alongside the reference ITR only, p40, SFFVrv-p40, and hSYNrv-p40 constructs in neuroblastoma derived SH-SY5Y cell line known for partial hSYN promoter activity^44^ and the non-neuronal HeLa cell line without detectable hSYN promoter activity.^41^ SH-SY5Y cells showed similar levels of GFP expression from all the p40 hybrid promoters tested, indicating a low influence of the NRSE on expression in these cells (Fig. 5b), as previously described.^44^ In contrast, HeLa cells showed a significantly reduced expression when the NRSE was present compared with the same construct without the NRSE (Fig. 5c; SFFVrv-p40 vs. SFFVrv-NRSE-p40; hSYNrv-p40 vs. hSYNΔrv-p40; SFFVrv-p40 vs. hSYNrv-p40; and p40 vs. hSYNrv-p40).

Furthermore, to investigate which of the two constructs, the hSYNrv-p40-GFP or the SFFVrv-p40-GFP, would be able to support specific expression restricted to the neurons, and thus enable AAV library selection in preclinical models of the human central nervous system, we transduced iPSC-derived cortical organoids^26^ with each construct packaged in AAV-7m8. Specifically, we compared the constructs’ efficiency in GFAP-expressing glial cells and neuronal nuclear antigen (NeuN)-expressing neurons. Both constructs showed robust expression in neurons (Supplementary Fig. S7c–e and Fig. 5f).

As expected, SFFVrv-p40 was able to express in glial cells as well as neurons (Fig. 3d–f and Supplementary Fig. S7d, 62.6% ± 23.8% of glial cells). At the same time hSYNrv-p40 was 7.9-fold less efficient at GFP expression in glial cells compared with hSYNrv-p40 expression in neurons (Fig. 5f, 12.4% ± 4.8% of glial cells). While GFP expression from the hSYNrv-p40 was still detected in some GFAP-positive cells (Fig. 5e, f), it was 5.1-fold weaker than the GFP expression driven by SFFVrv-p40 in GFAP-positive cells (Fig. 5f), thus confirming the hypothesis that the ubiquitous activity of p40 can be limited to specific cell types by incorporating a strong silencing element such as RE-1/NRSE (Fig. 5d–f).

## DISCUSSION

In light of recent adverse events and fatalities in gene therapy trials,^7^ efforts to improve vector safety are more important than ever. For clinical benefits to be achieved, vectors must enable the functional expression of their delivered cargo rather than delivery only.^7,45^ Therefore, the measure of the EXI should be considered when characterizing the functionality of a capsid when comparing well-performing candidates. Using a capsid that reliably converts the DNA cargo into RNA message in desired target cells allows achieving the therapeutic efficacy at a lower vector dose, increasing safety and lowering the cost of therapy.^7^

Over the years, many novel approaches to develop AAV variants via directed evolution have been developed to include effective cell entry, uncoating, and expression of the capsid transgene as a part of the selection process.

Some of the recent approaches added to the AAV bioengineering toolbox include spatial separation of library injection and recovery,^46^ use of Cre-recombinase in ubiquitously CRE-expressing mice (CREATE)^17^ as well as in mice with cell-type-specific CRE-recombinase expression (M-CREATE),^18^ *in vitro* Cre-recombinase shortening of the platform to link a random barcode to an inserted fragment in the capsid (BRAVE),^47^ introduction of Cre-recombinase alongside the capsid library into animals with loxP-flanked reporter genes (iTransduce),^19^ and a bicistronic platform allowing sorting for reporter-positive cells.^20,21^

However, while potentially robust in *in vitro* settings or when using transgenic animals, platforms relying on transgenic Cre-recombinase or reporters do not enable selections in primary cells and tissues, including human cells. On the contrary, most platforms that recover the AAV candidate sequences from DNA—even after sorting for reporter-positive cells—suffer a risk of coselecting for “passenger” AAVs. The “passenger” AAVs are variants that entered reporter-positive cells, but do not contribute to transgene expression due to their inability to overcome multiple possible intracellular hurdles, such as nuclear translocation,^48^ failure to uncoat inside the nucleus,^49,50^ or inefficient second-strand synthesis.^51^

Platforms allowing the recovery of capsids expressed directly from a CMV promoter have also been developed.^13,52^ While these overcome the abovementioned limitation and allow for capsid sequence expression in the target cells, including primary cells, they suffer from substantial manufacturing limitations,^13,52^ similar to those observed with our HTE platform (Supplementary Figs. S1 and S2 and Supplementary Results section). Another method of directly linking transgene expression to a capsid variant is the BRAVE approach.^47^ However, this approach would require complicated and potentially error-prone long-read sequencing if used to select capsid libraries based on DNA shuffling, domain swapping, or random mutagenesis.^47^

On the contrary, the recently published TRACER platform allows recovery of full-length expressed capsid sequences from target cells. While TRACER in principle offers an alternative approach to the herein presented platform, it suffers from reduced packaging efficiency. The impaired packaging can most likely be attributed to the coexpression of the capsids from the non-AAV promoter and the native p40 promoter during production, given that the use of nonAAV promoter for *cap* expression has previously been shown to be deleterious for packaging.^22,52^

The suboptimal packaging does not affect our FT platform as the capsid expression during the production is solely driven by the AAV native p40 promoter (Supplementary Fig. S1 and Supplementary Results section “Packaging”). However, it should also be noted that the most recently published DELIVER platform is able to achieve good packaging efficiencies despite not utilizing the endogenous AAV-p40 promoter.^23^

While the authors did not compare the packaging efficiency of their DELIVER platform to the natural rep-cap configuration to fully understand the effect their muscle-specific promoters had on packaging, their results suggest that some exogenous promoters may support high AAV production efficiency and high selective strength of the platform without depending on the AAV-p40 promoter.

Of note, the TRACER platform enhances cell-type-specific selection by utilizing a cell-type-restrictive promoter rather than the endogenous p40 promoter to drive capsid gene expression. However, in the light of our data showing the activity of p40 in multiple cell types *in vitro* and *in vivo*, it remains to be tested whether the TRACER platform can indeed select for AAV capsids that only work in the target cell type. Unwanted capsids expressed by the ubiquitous p40 promoter in off-target cell types could contaminate capsid product expressed from the cell-type-specific promoter, making the overall selection process less specific.

This may have occurred in the original publication as the authors found that capsids selected from the GFAP promoter-driven library were not transducing GFAP-expressing astrocytes.^22^ In contrast, the human synapsin promoter is very efficient at silencing the activity of p40 in nontarget cells as shown by hSYN-TRACER results^22^ as well as in our current data (hSYNrv-p40, Fig. 5).

The p40 promoter on its own, or in combination with binding sites from the bidirectional transcription factor from the SFFV promoter, was able to ubiquitously drive RNA expression in HeLa, HEK293T, HuH-7, HepG2, and A549 cell lines, and in primary fibroblasts (Fig. 4b), as well as in primary human hepatocytes *in vivo* (Fig. 2a) and in all *in vivo* mouse tissues evaluated (Fig. 4d). It may be surprising that p40 was functional in the absence of helper virus and AAV Rep2 proteins.^25,53^ However, the capsid expression regulation depends on the type of *rep2* deletion^25^ and could also be influenced by the type of target cell.^53^ Overall, the mechanism of p40 activation and the interplay with other promoter elements are interesting observations that will be investigated further in subsequent studies.

While additional studies are required to fully understand the p40 promoter, the herein-reported observations allowed us to form some hypotheses regarding the inactivation of p40 by silencing elements. Regarding the SFFVrv-p40 construct, there is ample published evidence that the SFFV promoter is prone to silencing in primary human hematopoietic stem cells.^54^ Whether this is the underlying mechanism of the reduced SFFVrv-p40 activity seen in some murine tissues (Fig. 5a) remains unknown. The mLSP has no known silencing factor binding sites, and we did not observe significant reductions in the p40 activity when linked to mLSPrv in any of the analyzed tissues (Fig. 5a).

In addition, mLSPrv-p40 did not exhibit a robust liver-specific improvement of p40 activity, despite having several liver-specific enhancing transcription factor-binding sites.^38,39^ The most substantial impact on the activity of p40 was exerted by hSYNrv, which substantially reduced p40 activity in all cells but neurons (Fig. 5f). We hypothesize that this was most likely due to the RE-1/NRSE, which is known to silence mRNA expression in cells that express the RE-1 silencing transcription factor/neuron-restrictive silencing factor (REST/NRSF).^55,56^ This element is expressed in every mammalian cell but rapidly degraded in neurons, leading to a neuron-restrictive expression profile of the hSYN promoter and other promoters incorporating the RE-1/NRSE.^55,56^

In summary, we showed that the SFFVrv-p40 promoter could support library selection at the RNA level *in vitro* (in HuH-7 cells) and *in vivo* (in primary human hepatocytes in the FRG xenograft mouse). Our results indicate that the p40 promoter can express *in vitro* and *in vivo* when using the sequence containing the transcription factor binding sites previously identified in the 154 nt version of the promoter.^25^ An AAV2-p40 promoter activity comparison against the AAV1 and AAV8 versions of p40 has recently shown highest activity of the AAV2-derived promoter.^57^ Moreover, our findings also suggest the option of adding silencing transcription factors to guide the p40 activity to certain cell types (*e.g.*, RE-1/NRSE).

The RC library selection in the humanized FRG mouse model demonstrated that the top 5 enriched variants (together accounting for 84.8% of all NGS reads in the second round of RC selection) all performed similarly or worse than AAV2, indicating that the RC platform did not select for the most optimal capsid. Hence, the selection may be hampered by replication biases and cellular changes occurring upon Ad5 superinfection, removing certain cellular barriers (reviewed in Nonnenmacher and Weber^58^). Interestingly, the sixth-best variant selected using this platform (RC06, only accounting for 1.3% of all NGS reads in the second round of RC selection) performed better than all other selected variants at the RNA level (Fig. 2f).

While the selection of RC06 could be a random event, it does indicate that the RC platform may lead to the selection of highly functional variants and thus has its place in the AAV bioengineering toolbox. In contrast, the capsids selected using the FT-RNA platform performed well in human hepatocytes (Figs. 2 and 3), presumably because the RNA-based selection (Fig. 1b) favors variants that can drive high levels of transgene expression.

The IF staining results, which due to animal-to-animal variability may be difficult to interpret, overall confirmed the results obtained using NGS analysis of the EXI, highlighting the power of NGS-based analysis, as, for example, FT11 (Fig. 3f) shows brighter GFP than NP59 when using mice with the same RI (Fig. 3g and Supplementary Table S2). Specifically, the NGS-based approach allows for high-throughput comparison of multiple vectors in the same animals, removing any potential animal-to-animal variability.^29^ The EXI guides identification and ranking of variants that drive an overall similar RNA expression.

Using NGS, we showed that vectors selected with the FT platform (*i.e.*, AAV-FT01, AAV-FT04, and AAV-FT11) were, on average, more efficient at functionally transducing human primary hepatocytes *in vivo* than variants identified using the canonical RC approach (*e.g.*, AAV-RC01) (Fig. 2d). Importantly, IF staining revealed that RC01, RC06, FT01, FT04, and FT11 were specifically transducing human cells rather than mouse hepatocytes (Fig. 3b–g). Furthermore, our data using AAV-NP59 clearly indicate that to accurately compare the expression of multiple AAV variants in the hFRG preclinical model of the human liver, it is critical to use mice with similar engraftment levels (Fig. 3g vs. Supplementary Fig. S5g).

The situation is even more complicated when evaluating results reported by different research teams, which may also use different transduction, harvest, and analysis protocols, as well as measure liver repopulation with human cells. In summary, it is our opinion that to correctly compare multiple vectors in complex models, such as xenograft mice, it is vital to complement the NGS analysis of multiple variants in the same animal with individual testing using an alternative readout such as IF staining, while also ensuring that the animals have highly similar levels of repopulation.^35^

## CONCLUSION

Overall, based on our results and published data, we identified several characteristics highlighting the strengths and weaknesses of some of the different AAV selection platforms (summarized in Table 1). First, the studies with the HTE platform confirmed that using an exogenous promoter lowers vector packaging efficiency, but does not affect the vector cross-packaging. In contrast, the p40-driven capsid expression in the FT platform allows robust packaging with minimal cross-packaging, improving the quality of the final library. The FT platform also allows for reliable capsid expression in the target cells, enabling stringent selection of highly functional variants from vector-encoded RNA.

**Table 1.** Summary of library platform performances

Measure	RC		FT		HTE	
Manufacturability	++		++		−	
Cross-packaging	++		+		+	
Expression of cap and reporter	n/a		++		− (vHTE)	++ (eHTE)
Selection stringency	++ (+Ad5)	− − (DNA)	+ (DNA)	++ (RNA)	− (DNA)	− (RNA)
Candidate performance in this study	+		++		n/a	
Dependence of outside factors	Cells/tissue of interest must be permissive for helper virus infection		None		None	
Potential skewing	Highly replicating variants may be favored		No known skewing		Strong skewing, unknown mechanism	
Mutagenesis of capsids during selection	Yes, in the presence of Ad5 infection^4^		No, unless error-prone polymerase is used for capsid recovery		No, unless error-prone polymerase is used for capsid recovery	
Sorting cells from complex tissues	No (+Ad5)	Yes (DNA)	Yes		Yes	

Performance grades: ++ = highest; + = adequate; − = suboptimal; − − = lowest.

Ad5, adenovirus 5; FT, functional transduction; HTE, high targeted expression; RC, replication competent.

Compared with the similarly stringent RC-Ad5 method, the FT-RNA selection does not depend on outside factors, such as helper virus permissiveness, which limits the utility of the RC platform to permissive target cells. Being replication incompetent, the FT platform is unbiased toward variants that can replicate with higher efficiency in the presence of Ad5 despite the lower overall transduction efficiency. In contrast, the RC-Ad5 approach allows for viral replication-based mutagenesis of the cap genes during the selection, which is most likely caused by Ad5.^4^ We have shown previously that this inherent mutagenesis step allows the variants to rapidly adapt and acquire positive characteristics.^4^

While such mechanisms do not apply to the PCR-rescue-based platforms (*i.e.*, FT, TRACER, and CREATE), the use of error-prone polymerases during the library recovery step can be implemented to increase library variability as previously reported.^16^ Lastly, a compelling feature of the FT platform, which does not apply to the RC platform, is the ability to complement the selection process by sorting a specific cell subpopulation from complex tissues or organoids using cell-type-specific markers. This allows for simultaneous individual selections to be performed in various cell types present in the selection model or to enrich for the target cells enhancing the overall selection process.

As the FT-RNA selection method has many of the aforementioned benefits, and on average the novel variants identified using the FT platform in this study perform better than the variants selected using the RC-Ad5 strategy (Fig. 2d), we hypothesize that the FT-RNA platform has the potential to become a powerful tool in the AAV bioengineering toolkit and may support the development of novel variants for preclinical and clinical applications.

## Supplementary Material

Supplemental data
