# Supplementary material for: AAV-p40 Bioengineering Platform for Variant Selection Based on Transgene Expression
Source: Hum Gene Ther. 2022 Jun 10;33(11-12):664–82. doi: 10.1089/hum.2021.278 (PMC10112876; doi:10.1089/hum.2021.278)
Supplement: Supplemental data [file Suppl_Data.docx]

**Supplementary information**

**Including:**

- **Supplementary results, discussion, methods, and relevant references**
- **Supplementary figures**
- **Supplementary tables**

**Supplementary results**

**Selection platform designs**

The Replication Competent (RC) platform was based on the wild-type (wt)AAV2 genome configuration and was previously successfully applied to support development of novel AAV capsid variants by us and others.^1,2^ Using this platform, AAV variants can be selected either by super-infection with a helper virus^3^ or PCR-amplification of DNA delivered to the target cells^4^ (Fig. 1a). However, we hypothesized that due to the lack of control of the viral replication process, including target cell selection in complex tissues, and the inability to control the intracellular location of templates for PCR amplification (for PCR-based amplification approach), the RC platform does not support stringent selection and suffers from a high risk of selecting suboptimal vector candidates. We speculated that a platform where the *cap* gene would be the expressed transgene in the target cells could gear selection towards highly expressing vectors when combined with the directed evolution approach. The RC platform follows the wtAAV2 genome configuration, with all three open reading frames of the *cap* gene being expressed from under the endogenous p40 promoter.^5^ Thus, unfortunately, the RC platform cannot be used for selection based on *cap* gene expression, since the p40 promoter has reportedly low transcriptional activity^6^ potentially due to the presence of Rep proteins and absence of helper virus proteins,^7^ and can also be inactive depending on the cell type.^8^ To overcome this, we have re-engineered the original RC selection platform to introduce an exogenous promoter and a reporter gene (Fig. 1).

In the first variation, the resulting platforms contained a GFP expression cassette driven by a spleen focus-forming virus (SFFV) long terminal repeat promoter^9^ cloned in reverse orientation to the p40-*cap* in place of the *rep* coding regions upstream of the p40 promoter (Fig. 1b). Of note: SFFV-GFP can be replaced with any reporter cassette, provided it fits within the packaging capacity of an AAV vector. This system allows for selecting vector candidates based on functional transduction as measured by GFP expression and was, therefore, named Functional Transduction (FT) platform.^10^ Following transduction of target cells, this platform allows for the sorting of green fluorescent protein (GFP)-expressing cells followed by DNA extraction of vector genomes from transduced cells (as previously described^10^). We also hypothesized that the SFFV-p40 hybrid promoter and other p40-hybrid combinations might enable *cap* gene expression in the target cells, as hypothetical downregulating elements present in *rep*2 may have been removed when the rep gene was truncated to make space for the GFP expression cassette.^7^ Consequently, this allowed for capsid recovery from RNA/cDNA extracted from successfully targeted cells, as reported recently.^11^

In an attempt to improve RNA-based capsid recovery, we designed also another platform termed High Targeted Expression (HTE) (Supplementary Fig. 1a), which expressed the capsid gene library from the strong and ubiquitous SFFV promoter. To enable the selection of functionally transduced cells prior to capsid recovery, a GFP reporter gene was linked to *cap* via an internal ribosomal entry site (IRES). Two different IRES elements were evaluated, a short version of the virus-derived (vHTE) encephalomyocarditis virus (EMCV) IRES^12^ and a eukaryotic IRES (eHTE) from the human early initiation factor 4G1 (eIF4G1)^13^ gene. Those two selection platforms follow the same selection methods as the FT platform. The main difference is that the retrovirus-derived SFFV promoter used in the HTE platforms has broad activity in many target tissues.^14,15^ In contrast, the activity of the p40-hybrid promoter present in the FT library is untested, making the HTE platforms theoretically more reliable to express the capsid gene in target cells.

**Packaging**

We validated each selection platforms based on their ability to support efficient vector packaging. This is critical for the HTE platforms (Supplementary Fig. 1a) since the non-AAV-native SFFV promoter could affect capsid protein expression, assembly, and/or encapsidation.^16,17^ Given that the Functional Transduction (FT) platform (Fig. 1b) uses the native AAV-p40 promoter to drive capsid expression, we anticipated it would support packaging efficiency similar to that of recombinant AAVs. The well-established RC platform (Fig. 1a) was used as a positive control.

To test the packaging efficiency, we cloned AAV *cap2*, *cap8,* and *capDJ* (encoding for capsid of AAV2, AAV8, and AAV-DJ^1^, respectively) into each of the platforms in place of the capsid library. As outlined in Supplementary Fig. 1b, the specific constructs required during the packaging reaction differed between individual platforms (see the Methods section for details).

AAV2 provided the highest yield in the context of the RC and FT platforms with median packaging at around 1 × 10^10^ and 3 × 10^10^ Benzonase and DNase-resistant vector genomes (vg) in crude lysates per 15 cm dish (resulting in 500 and 1,500 vg per producer cell, respectively). The same capsid produced with significantly lower efficiency in the two HTE platforms (Supplementary Fig. 1c). AAV8 produced nearly 10× more efficiently than AAV2 in the respective platform contexts with eHTE packaging with significantly lower efficiency than the RC reference platform. Finally, RC-encoded AAV-DJ capsid was packaged with efficiencies between those observed for AAV2 and AAV8, overall showing no significant difference between packaging efficiencies in the context of the RC, FT, and eHTE platforms. However, in contrast to the other two AAV variants, AAV-DJ packaged most efficiently in the vHTE platform. The results demonstrate that the packaging efficiencies with the vHTE and eHTE platforms were highly variable and capsid dependent. On the other hand, the FT platform consistently packaged at levels similar to the wildtype-like RC platform and showed the lowest overall variability compared to the other platforms (Supplementary Fig. 1c).

As the eHTE platform provided the lowest vector packaging efficiency in the context of the capsids tested and the packaging in the vHTE platform was highly variable, we evaluated if the packaging efficiency could be improved by optimizing expression levels of AAV2 Rep proteins. Therefore, we used a modified Rep2 construct reported to drive decreased levels of Rep78 and Rep68 expression caused by substitution of the ATG start codon with a less efficient ACG start codon.^18^ To this end, FT, vHTE, and eHTE constructs were used for packaging AAV2, AAV8, and AAV-DJ particles using either the previously used wild-type Rep2ΔCap helper plasmid or a mutated Rep2-ACG-ΔCap following the production scheme as outlined in Supplementary Fig. 1b (n=3 for each platform/capsid/Rep2 combination). In contrast to what we anticipated; all three capsids were packaged with substantially lower efficiencies in the context of the three platforms when packaged with the mutated Rep2 (Supplementary Fig. 1d-f). While none of the observed differences were statistically significant, we excluded Rep2-ACG from further experiments based on the observed lack of packaging improvement.

Due to the variability in packaging efficiency observed for the HTE platforms harboring the SFFV promoter-driven *cap*, we further investigated the effect of SFFV in the context of the FT platform. This is critical because in the FT platform the promoter that drives GFP expression is adjacent to the p40 promoter driving *cap* and, therefore, could influence *cap* expression (Supplementary Fig. 1g). Interestingly, no difference in the AAV-DJ packaging was observed when the SFFV promoter was replaced with a liver-specific promoter (LSP, apolipo protein E [ApoE] enhancer/ human alpha anti-trypsin [hAAT] promoter)^10^, a neuron-specific promoter (hSyn, human synapsin)^19^, or a cardiac and smooth muscle-specific promoter (MLC, ventricular myosin light chain 250 nt version)^20^. This indicates that the steps involved in packaging were not influenced by a secondary promoter (Supplementary Fig. 1h).

**Cross-packaging**

We next investigated whether the novel capsid selection platforms could reliably package the AAV *cap-*encoding genomes into the corresponding capsid particle (Supplementary Fig. 2a), in contrast to cross-packaging (Supplementary Fig. 2b). To this end, we performed AAV cross-packaging experiments using the four selection platforms transfecting a functional Cap2 and a mutant Cap2 with a Y576* deletion, which prevents the formation of functional particles.^16^ The AAVs were produced with a 1:1 mix of the Cap2/Cap2 Y576* plasmids at a dilution series ranging from 25,000 to 500 copies of library plasmid per transfected cell in individual 15 cm dishes.

Lowering the amount of the capsid-containing library plasmid per packaging cell decreased the frequency of cross-packaging, as expressed by the percentage of Cap2 Y576* genomes detected in Benzonase and DNase-resistant fully assembled AAV particles measured using Illumina-based amplicon-seq next-generation sequencing (NGS). Cross-packaging was the most frequent and reached 25-30 % in all platforms when produced with the highest library plasmid input (Supplementary Fig. 2c) but in agreement with previously reported data^17^ could be reduced to under 5 % by lowering the library plasmid input to 500 copies per cell. This effect of decreased cross-packaging with the decreased amount of input plasmid was observed in all platforms. The effect was most prominent for the AAV2 wild-type-like RC platform, followed by the FT and both HTE platforms (Supplementary Fig. 2c).

Importantly, as shown in Supplementary Fig. 2d, lower plasmid input did not significantly affect the overall vector titer. This indicated that the libraries could be produced at all conditions tested and confirmed that the production efficiencies were higher using platforms in which capsid expression was driven by the native p40 promoter (RC and FT), rather than a non-AAV promoter, as reported previously.^17^

**Mini-library selection.**

As transgene expression is the ultimate goal for most canonical gene therapy applications, we designed an experiment that would allow us to distinguish which of the selection platforms tested is the most efficient at identifying the most functional variant(s) from the library mix.

Specifically, we packaged and performed a selection of a mini-library composed of three well-defined AAV variants, namely AAV2, AAV8, and AAV-DJ, in the human hepatocellular carcinoma cell line HuH-7. AAV2 and AAV-DJ have similar and strong tropism for HuH-7 cells, while AAV8 is a weak performer in those cells^1,21^, allowing us to test the stringency of the individual selection platforms. A validation study in which HuH-7 cells were transduced with an equimolar mix of the three AAV variants encoding barcoded transgenes confirmed that AAV2 and AAV-DJ had very similar efficiency at cell entry (DNA reads). However, AAV-DJ was a better overall performer based on higher transgene expression (RNA/cDNA reads) (Supplementary Fig. 3a). Conversely, AAV8 transduced HuH-7 cells with low efficiency based on DNA and RNA/cDNA reads. Thus, we hypothesized that the most functional library platform would positively select AAV-DJ and select against AAV8 when tested on HuH-7 cells.

The three AAV variants were packaged in each of the library constructs and were subsequently mixed at equimolar ratios to create the four individual libraries (Supplementary Fig. 3b) and used to transduce HuH-7 cells. We used a universal oligo pair to amplify homologous capsid regions containing sequences unique for each capsid gene that served as unique NGS barcodes (Supplementary Fig. 3c).

As the RC platform allows for viral replication in the presence of a helper virus, conditions for optimal replication of the RC.2-8-DJ library were determined using qPCR analysis of cells infected with the library in the presence and absence of Ad5-driven library replication, as previously reported.^22^ We detected strong replication at multiplicities of infection (MOIs) as low as 50 vg/cell in the presence of wild-type human Adenovirus 5 (wtAd5) (RC-low-Ad5). The optimal multiplicities of transduction (MOTs) for the FT and HTE platforms were determined by evaluating the GFP signal in cells transduced with the respective 2-8-DJ libraries. We identified a dose that would lead to transduction levels below 30 %, which allows for efficient sorting of GFP-positive cells at the overall low library input, which should minimize the selection of non-functional “passenger” variants. A dose of 500 vg/cell gave the desired level of transduction of 15-20 % of GFP positive cells for the FT and eHTE platforms, as this level lowers the risk of cells being saturated with AAVs, while still allowing to efficiently sort enough cells to extract required amounts of DNA and RNA for downstream analysis. Interestingly, the vHTE platform did not lead to a detectable level of GFP expression, suggesting that the EMCV IRES used^12^ was not functional in the current construct configuration. Therefore, we performed the vHTE selection using unsorted cells. To allow for direct comparison between RC and the other platforms, the DNA recovery of the RC selection was performed at the MOI of 500 vg/cell without super-infection with Ad5 (Supplementary Fig. 3b, RC-high-DNA). In addition, to enable the evaluation of RC under the AAV replication conditions, cells were infected with the library at an MOI of 50 (low, see above) and 500 (high) vg/cell and super-infected with wtAd5 (Supplementary Fig. 3b, RC-low-Ad5 and RC-high-Ad5). Selection using the FT and HTE platforms was performed by recovering capsid sequences from DNA (FT/vHTE/eHTE-high-DNA) and RNA (FT/vHTE/eHTE-high-RNA) (Supplementary Fig. 3b).

After just one round of selection using the RC platform in the presence of wtAd5, we found that AAV-DJ was the most highly selected AAV variant and accounted for 71.8 % and 82 % of reads at the RC-low-Ad5 and RC-high-Ad5, respectively (Supplementary Fig. 3b). Interestingly, the variance between n=7 independent selection experiments was lower for RC-high-Ad5 than RC-low-Ad5, suggesting that an optimal dose of wtAd5 should be identified when working with RC libraries to enhance the selection process, as previously described.^23^ Recovering the RC library from DNA in the absence of Ad5 also led to a positive selection in favor of AAV-DJ. However, the selection was substantially weaker than in the presence of wtAd5 (Supplementary Fig. 3b, 47.5 % for RC-high-DNA vs. 82 % for RC-high-Ad5), highlighting the positive impact of AAV replication on the selection of functional variants that complete the intracellular journey from cell entry to the nucleus in an experiment using the three chosen capsids.

The capsid selection using the FT platform following cell sorting based on GFP signal prior to DNA and RNA extraction also allowed for substantial enrichment of AAV-DJ, which accounted for 71.4 % of detected reads at the DNA and 80.2 % at the RNA level (Supplementary Fig. 3b). These data suggest that when screening AAV libraries for the most functional variant, selection at the RNA level is more stringent than selection at the DNA level.

As the vHTE platform did not enable sorting for GFP-positive cells, there was no positive selection for AAV-DJ over AAV2 when analyzing DNA from the vHTE platform (44 % each), yet AAV2 was selected at the RNA level (49 %), which corresponded to a reduction of the AAV8 reads. Selection using the eHTE platform showed very similar results but with a lower contribution of AAV8 when performing AAV recovery from the DNA and RNA extracted from GFP-positive cells (Supplementary Fig. 3b).

**Activity of the native AAV-p40 promoter**

We aimed to identify a method to enable capsid recovery from expressed RNA. However, the HTE platforms, which we hypothesized would be the most efficient at reliably expressing the *cap* gene in a broad range of cell types, were substantially less efficient in their ability to reliably package transgenes. Therefore, we wanted to establish whether the FT platform could be used to express transgenes and, as such, the capsid mRNA of highly efficient AAV variants in target cells. We first performed transfections using *cap2* in the FT and vHTE platforms in the absence and presence of the Rep2 and pAd5 helper plasmids (Supplementary Fig. 6a). Capsid gene expression levels were measured using RT-qPCR of *cap2* and normalized to endogenous human β-actin. No detectable difference between the *cap2* expression was observed between the FT and vHTE platforms with and without helper plasmids (Supplementary Fig. 6b). Furthermore, to determine if p40 on its own could express a transgene without helper genes, or if this activity required proximity to SFFV, we generated multiple control constructs (Supplementary Fig. 6c). We chose a self-complementary (scAAV) configuration for these constructs to minimize any potential bias due to AAV genome processing in the assessment of the promoter activities. Specifically, as AAV second strand synthesis has been shown to be a barrier in AAV transgene expression we hypothesized that removing this rate-limiting step from the analysis, especially since the second strand synthesis could be different depending on the target cell type, would increase quality of the data.^24^ Specifically, we compared a construct containing only the p40, SFFV-reverse-p40 (SFFVrv-p40, the same configuration as used in the FT platform), and SFFVrv promoters. Constructs containing the strong ubiquitous cytomegalovirus (CMV) enhancer/chicken beta-actin promoter/rabbit globin intron (CAG) promoter and the very weakly expressing D region of the AAV ITRs as well as untransduced cells were included as positive and negative controls, respectively. All constructs were cloned into a self-complementary AAV plasmid upstream of a GFP reporter gene (scAAV-‘promoter’-GFP), and were packaged into the AAV-7m8^4^ variant known to be highly functional in a variety of *in vitro* and *ex vivo* models.^21^ Following transduction at a multiplicity of transduction (MOT) of 1,000 vector genomes (vg) per cell (vg/cell), we analyzed the GFP mean fluorescent intensity (MFI) (Supplementary Fig. 6c). While none of the promoters drove GFP expression to the same extent as CAG, cells transduced with p40 and SFFVrv-p40 vectors expressed GFP at MFIs higher than the ITR construct (Supplementary Fig. 6d). Collectively, we demonstrated that the 154 nt p40^5^ in the FT construct can induce transgene expression that is potentially boosted by the adjacent reverse-orientation SFFV promoter. To expand the testing strategy, we added unique barcodes (BC) to the 3’UTR in each construct to allow for an NGS-based analysis of transgene expression (Supplementary Fig. 6e). Control studies showed that inclusion of the barcode did not significantly affect GFP expression as compared to non-barcoded constructs (Supplementary Fig. 6f). The only exception was the slightly reduced expression strength of the CAG-GFP construct, which resulted in a relative increase of the p40- and SFFVrv-p40-driven GFP expression construct (Supplementary Fig. 6f).

## Supplementary Discussion

Capsids assembled just as well in the context of the FT platform as they did in the RC platform, most likely due to the use of the p40 promoter in the FT platform. The decrease in packaging efficiency observed in the context of a non-AAV SFFV promoter and an IRES in the selection platform constructs may potentially have been caused by suboptimal interactions of the packaging process (such as spatial or temporal precision) with *rep2* or Ad5 helper genes. This hypothesis is emphasized by recent findings by Ogden *et al.* (2019)^16^, who tested AAV2 substitutions and insertions for packaging in a pCMV and a pRep2 backbone. The authors showed that there might be fewer deleterious mutations (including stop codons) affecting the same capsid in the pCMV than in the pRep2 context.^16^ Schmitt *et al.*,^17^ reported a drop in packaging efficiency when using the pCMV platform in the context of cross-packaging. Moreover, using an IRES element in the HTE platform, it was necessary to mutate the original AAV polyadenylation signal to ensure that the RNA incorporated the capsid coding region, the IRES, and the GFP reporter. This modification could also negatively affect the capsid assembly.

Interestingly, the reduction in yield by using the ACG-Rep2 has recently been published and expanded for other capsids and *rep* genes.^25^ This confirms our observations and also indicates that the original finding was false or that some discrepancies in the AAV production lead to the discrepancies shown in this manuscript and published elsewhere.^25^

The RC platform selection confirmed that a lower MOI condition (RC-low-Ad5) provided less reliable results than the RC-high-Ad5 condition, as previously reported.^23^ However, our observations that the selection process was more reliable when recovering sequences from the replicated virus than DNA are not in agreement with previously published reports.^23^ As discussed earlier, the selection from replicating viruses may favor variants that can replicate with high efficiency but will not lead to high transgene expression in the absence of the helper virus.^26,27^ The mini-library (2-8-DJ) may not have shown this disadvantage of the replicating system as the replication efficiencies of the three variant were not distinct enough. Other studies that have utilized a larger number of variants to study this phenomenon have reported preferential selection of variants based on their replication rather than improved performance.^23^ Moreover, AAV-DJ was selected using the RC system in the presence of wtAd5 and might, therefore, be very good at replication and transduction, thus not showing the replication bias.

**Supplementary Materials and Methods**

**Plasmid preparations.** The Replication-Competent (RC) library selection platform was a generous gift from the Kay lab (Stanford University, CA, USA) used in several previous studies.^22,28,29^ It almost entirely resembled the wtAAV2 genome, retained the native SwaI restriction site just upstream of the VP1 start, and only had the native blunt SnaBI restriction site changed to a 3’-overhang producing NsiI site for ease of library and capsid cloning, all of which are flanked with SwaI and NsiI restriction sites as well. All restriction enzymes and the T4 DNA ligase (used in all ligation steps of this manuscript, Cat# M0202) were purchased from New England Biolabs (NEB). All primers for cloning, next-generation sequencing (NGS), and other PCRs can be found in Supplementary Table 3.

The various Functional Transduction (FT) plasmids were all based on the previously described FT-LSP platform.^10^ The reverse promoter driving GFP was changed using the NotI and MluI sites flanking the LSP promoter. This allowed for the alternative promoters to be cloned into the construct to be flanked by an SV40 intron oriented towards GFP and the Rep2-p40 promoter oriented towards the capsid expression. SFFV was amplified from pCVL (Addgene, Cat# 50434) using SFFV_MluI_F and SFFV_NotI_R (Supplementary Table 3). hSyn was amplified from an Addgene plasmid (Cat# 26973) using hSyn_MluI_F and hSyn_NotI_R (Supplementary Table 3). MLC was amplified, using the MLC_MluI_F and MLC_NotI_R primers (Supplementary Table 3), from a lentiviral plasmid bearing the promoter^20^, which was a kind gift from Eddy Kizana (Westmead Medical Research Institute, Sydney, Australia). All versions of the FT plasmid had the same SwaI and NsiI restriction sites for ligation of capsids and capsid libraries.

The vHTE platform was synthesized by Genewiz Genomics (Suzhou, China) as a fragment containing EcoRV-SFFV-SV40intron-SwaI-50nt stuffer-NsiI-EMCV IRES-MfeI-eGFP-HSV TK pA-EcoRV signal. The EcoRV sites were used to move the construct into an ITR-containing plasmid with TMP resistance. The SwaI and NsiI sites allowed insertion of capsids and capsid libraries, and the NsiI and MfeI sites were used to change the IRES. The eIF14G1 IRES was amplified from HuH-7 cell cDNA using previously published primers^13^ but adding restriction sites for NsiI (eIRES_NsiI_F) and MfeI (eIRES_MfeI_R). To create a working IRES, the AAV capsids native polyadenylation signal between the stop codon and the NsiI (SnaBI in native wtAAV2 configuration) site were mutated from ‘AATAAA’ to AAGCGA’ using overlapping mutagenesis primers (mut_pA_1 and mut_pA_2, Supplementary Table 3), amplifying the entire pRep2Cap2/8/DJ packaging plasmid and reconstructing the mutated plasmid using NEBuilder assembly (NEB, Cat# E2621).

Self-complementary AAV plasmids (scAAV) were all based on pscAAV-CAG-GFP (Addgene, Cat# 83279), which was a kind gift of Prof. Mark Kay. All promoter constructs were created by inserting the promoters into the AvrII and BamHI restriction sites flanking the CAG promoter, apart from the ITR-GFP construct, which was created by digesting the CAG-GFP using AvrII and BamHI, blunting (Q5 [NEB, Cat# M0491L], dNTPs [NEB, Cat# N0447] (72 °C for 10 min), and ligation. The Rep2-p40 was amplified using p40_AvrII_F and p40_BamHI_R and SFFVrv-p40 were amplified using SFFVrv_AvrII_F and p40_BamHI_R (Supplementary Table 3). Lastly, the p40 promoter was deleted in the pscAAV-SFFVrv-GFP constructs using MluI and BamHI followed by blunting and ligation as described above. All other promoter constructs were cloned this way with the corresponding primer sequence following the same strategy as stated above. All primers can be found in (Supplementary Table 3). Templates for the full-length LSP was the LSP-GFP-barcoded construct,^10^ the minimal LSP was taken from the FT-LSP platform^10^, and the hSYN was cloned from the FT-hSYN platform (see above). The short NRSE was introduced as a long overhang in a PCR primer and removed by amplifying the whole hSYNrv-p40-GFP plasmid with primers outside the NRSE followed by KLD (NEB, Cat# M0554) mediated re-ligation. The short indexing barcode was introduced to the respective scAAV-‘promoter’-GFP construct using NotI and StuI sites just downstream of the GFP stop codon.

Novel capsid variants selected in the peptide library screen from the RC selection (n=8) as well as the capsid FT11 were cloned using two primers binding to the pRep2Caplco2_SfiI plasmid and each bearing half of the peptide-coding region as a long overhang (see Supplementary Table 3, RC01-RC08_F/R, FT11_F/R). Next, the whole plasmid was amplified using the respective primers, reassembled using KLD enzyme mix (NEB, Cat# M0554), and transformed into bacteria. Finally, the remaining FT-selected capsids (FT01-10 & 12, n=11) were cloned using a universal reverse primer for all peptides and individual forward primers with very long 5’-extensions bearing the entire peptide sequence (see Supplementary Table 3, FT01-10,12_FTF/R, Universal_FT_R).

***In vitro* transfections and reverse transcriptase qPCR (RT-qPCR).** Transfection of single 15 cm dishes of AAV *cap2* in the FT and vHTE platforms was performed using PEI and either 5 µg of the FT/vHTE-SFFV-AAV2 (n=3) alone or including the pRep2 and pAd5 helper plasmids at a 1:1:2 molar ratio (n=3), as described for AAV production. RNA extraction and cDNA synthesis were performed as described below, 72 hours after transfection. For RT-qPCR, 2.5 µL of 1:10 diluted cDNA was used alongside the RT- control, using qPCR reagents described above. Primers for β-actin (b-actin_F/R) and *cap2* (wtCap2_F/R) (Supplementary Table 3) were used for amplification of housekeeper and target, respectively. Relative quantities (RQ) were calculated following: RQ = 2^-(Ct[cap2]-Ct[b-actin])^

**Mini-library selections.** The 2-8-DJ library selection was performed by mixing individually produced AAV2/8/DJ in the respective RC/FT/vHTE/eHTE platforms (see above) and infecting/transducing cells at various MOIs/MOTs. The HuH-7 cells (24-well, 100,000 cells/well) were infected with the RC.2-8-DJ at five different MOIs ranging from 500,000 to 50 in log10 steps. The RC selection followed protocols reported previously.^22,29^ In brief, two wells were infected per MOI and six hours after infection cells were rinsed with 1X DPBS, and one of each replicate was super-infected with 1 µL wtAd5 (American Type Culture Collection [ATCC], lot. no. 70010153, 1 µL of this lot. no. was previously determined to lyse 100,000 HuH-7 cells within 64-72 hours^29^), while the other replicate served as a non-replication control. After the Ad5 super-infected cells were fully lysed (64-72 h after infection), the Ad5-treated and untreated cells were collected, subjected to three rounds of freeze-thawing, the debris was removed by centrifugation (5,000 × g, 10 min, 4 °C) and the cleared supernatant was transferred to a fresh tube. Subsequently, 2 µL of the lysate underwent DNaseI and Proteinase K digestion, as described above. For the first biological replicate of the experiment, qPCR was performed on the DNaseI/Proteinase K-digested lysate using Rep2_F/R primers (Supplementary Table 3) and the protocol described above. A 100-fold increase in Rep2 signal in the Ad5-treated sample compared to the untreated sample receiving the same amount of AAV was defined as a productive AAV replication. All conditions tested showed such an increase or higher. Hence, the lowest condition (MOI 50) was defined as the condition of choice for this and the additional six biological replicates for the RC selection in the presence of Ad5. For all biological replicates (n=7), the DNaseI/Proteinase K treated lysate of the MOI 50 and 500 (explained below) conditions were used for NGS analysis to assess the contribution of each capsid before and after each selection (details see below).

The selection of the 2-8-DJ library in the FT/vHTE/eHTE platform was performed by transducing HuH-7 cells (6-well plate, 400,000 cells/well) using MOTs of 5,000, 500, and 50 vg/mL for the first biological replicate. The MOT 500 resulted in 15-20 % GFP-positive cells for the FT and eHTE conditions, which we deemed sufficiently low (see Results) to be sorted for DNA and RNA extraction (sorting for GFP-positive and DAPI-negative cells using FACS protocol described above). The vHTE did not show any GFP expression, and therefore, DNA and RNA were extracted from the unsorted MOT 500 sample. The following six biological replicates were only performed using the MOT 500 condition.

Due to the discrepancy in doses that led to the minimal replication (RC, MOI 50) and the minimal sortable GFP expression (FT/eHTE, MOT 500), the RC replication was also performed at MOI 500 (RC-high-Ad5). Furthermore, RC selection from MOI 500 was investigated using DNA extraction from Ad5-untreated and unsorted cells as described for the vHTE selection.

**Supplementary References**

1. Grimm D, Lee JS, Wang L et al. In Vitro and In Vivo Gene Therapy Vector Evolution via Multispecies Interbreeding and Retargeting of Adeno-Associated Viruses. Journal of Virology 2008 82(12): 5887.

2. Lisowski L, Dane AP, Chu K et al. Selection and evaluation of clinically relevant AAV variants in a xenograft liver model. Nature 2014 506: 382.

3. Müller OJ, Kaul F, Weitzman MD et al. Random peptide libraries displayed on adeno-associated virus to select for targeted gene therapy vectors. Nat Biotechnol 2003 21(9): 1040-1046.

4. Dalkara D, Byrne LC, Klimczak RR et al. In Vivo–Directed Evolution of a New Adeno-Associated Virus for Therapeutic Outer Retinal Gene Delivery from the Vitreous. Sci Transl Med 2013 5(189): 189ra176.

5. McCarty DM, Christensen M and Muzyczka N. Sequences required for coordinate induction of adeno-associated virus p19 and p40 promoters by Rep protein. Journal of Virology 1991 65(6): 2936.

6. Labow MA, Hermonat PL and Berns KI. Positive and negative autoregulation of the adeno-associated virus type 2 genome. Journal of Virology 1986 60(1): 251.

7. Tratschin JD, Tal J and Carter BJ. Negative and positive regulation in trans of gene expression from adeno-associated virus vectors in mammalian cells by a viral rep gene product. Mol Cell Biol 1986 6(8): 2884.

8. Mendelson E, Smith MG and Carter BJ. Expression and rescue of a nonselected marker from an integrated AAV vector. Virology 1988 166(1): 154-165.

9. Faller DV, Weng H, Graves DT et al. Moloney murine leukemia virus long terminal repeat activates monocyte chemotactic protein-1 protein expression and chemotactic activity. Journal of Cellular Physiology 1997 172(2): 240-252.

10. Cabanes-Creus M, Westhaus A, Navarro RG et al. Attenuation of Heparan Sulfate Proteoglycan Binding Enhances In Vivo Transduction of Human Primary Hepatocytes with AAV2. Mol Ther Methods Clin Dev 2020 17: 1139-1154.

11. Cabanes-Creus M, Navarro RG, Liao SHY et al. Single amino acid insertion allows functional transduction of murine hepatocytes with human liver tropic AAV capsids. Mol Ther Methods Clin Dev 2021 21: 607-620.

12. Bochkov YA and Palmenberg AC. Translational efficiency of EMCV IRES in bicistronic vectors is dependent upon IRES sequence and gene location. BioTechniques 2006 41(3): 283-292.

13. Wong ET, Ngoi SM and Lee CGL. Improved co-expression of multiple genes in vectors containing internal ribosome entry sites (IRESes) from human genes. Gene Ther 2002 9(5): 337-344.

14. Bandaranayake AD, Correnti C, Ryu BY et al. Daedalus: a robust, turnkey platform for rapid production of decigram quantities of active recombinant proteins in human cell lines using novel lentiviral vectors. Nucleic Acids Res 2011 39(21): e143-e143.

15. Wahlers A, Kustikova O, Zipfel PF et al. Upstream Conserved Sequences of Mouse Leukemia Viruses Are Important for High Transgene Expression in Lymphoid and Hematopoietic Cells. Molecular Therapy 2002 6(3): 313-320.

16. Ogden PJ, Kelsic ED, Sinai S et al. Comprehensive AAV capsid fitness landscape reveals a viral gene and enables machine-guided design. Science 2019 366(6469): 1139.

17. Schmit PF, Pacouret S, Zinn E et al. Cross-Packaging and Capsid Mosaic Formation in Multiplexed AAV Libraries. Mol Ther Methods Clin Dev 2020 17: 107-121.

18. Li J, Samulski RJ and Xiao X. Role for highly regulated rep gene expression in adeno-associated virus vector production. Journal of Virology 1997 71(7): 5236.

19. Schoch S, Cibelli G and Thiel G. Neuron-specific gene expression of synapsin I. Major role of a negative regulatory mechanism. J Biol Chem 1996 271(6): 3317-3323.

20. Barth AS, Kizana E, Smith RR et al. Lentiviral Vectors Bearing the Cardiac Promoter of the Na+ Ca2+ Exchanger Report Cardiogenic Differentiation in Stem Cells. Molecular Therapy 2008 16(5): 957-964.

21. Westhaus A, Cabanes-Creus M, Rybicki A et al. High-Throughput In Vitro, Ex Vivo, and In Vivo Screen of Adeno-Associated Virus Vectors Based on Physical and Functional Transduction. Hum Gene Ther Methods 2020.

22. Cabanes-Creus M, Ginn SL, Amaya AK et al. Codon-Optimization of Wild-Type Adeno-Associated Virus Capsid Sequences Enhances DNA Family Shuffling while Conserving Functionality. Mol Ther Methods Clin Dev 2019 12: 71-84.

23. de Alencastro G, Pekrun K, Valdmanis P et al. Tracking Adeno-Associated Virus Capsid Evolution by High-Throughput Sequencing. Human Gene Therapy 2020.

24. Salganik M, Aydemir F, Nam H-J et al. Adeno-Associated Virus Capsid Proteins May Play a Role in Transcription and Second-Strand Synthesis of Recombinant Genomes. Journal of Virology 2014 88(2): 1071.

25. Mietzsch M, Eddington C, Jose A et al. Improved Genome Packaging Efficiency of Adeno-associated Virus Vectors Using Rep Hybrids. Journal of Virology 2021 95(19): e00773-00721.

26. Büning H and Srivastava A. Capsid Modifications for Targeting and Improving the Efficacy of AAV Vectors. Mol Ther Methods Clin Dev 2019 12: 248-265.

27. Nonnenmacher M and Weber T. Intracellular transport of recombinant adeno-associated virus vectors. Gene Ther 2012 19: 649.

28. Paulk NK, Pekrun K, Zhu E et al. Bioengineered AAV Capsids with Combined High Human Liver Transduction In Vivo and Unique Humoral Seroreactivity. Molecular Therapy 2018 26(1): 289-303.

29. Cabanes-Creus M, Hallwirth CV, Westhaus A et al. Restoring the natural tropism of AAV2 vectors for human liver. Science Translational Medicine 2020 12(560): eaba3312.

**
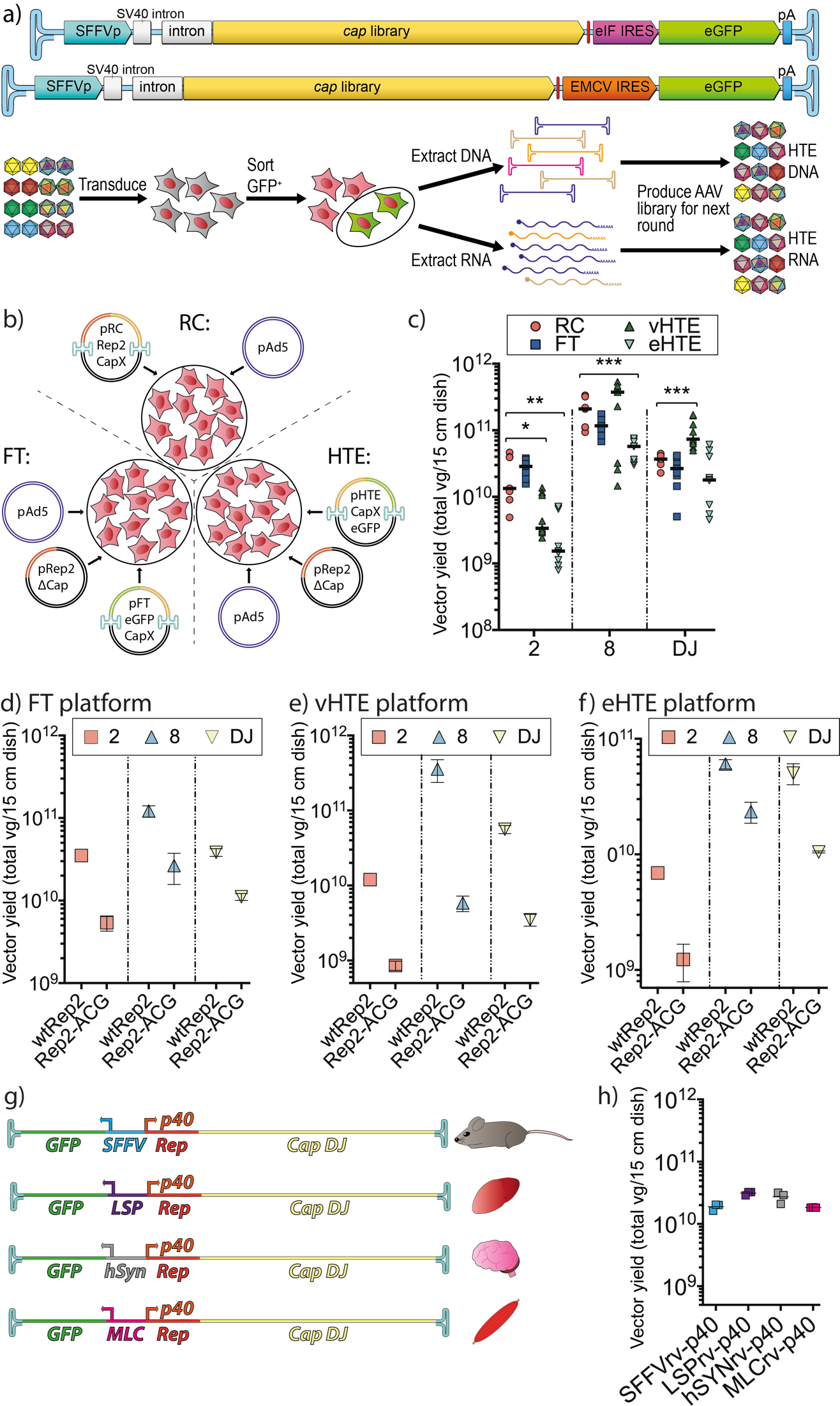
**

**Supplementary Figure 1. Manufacturability of AAV capsids using the Replication Competent (RC), Functional Transduction (FT), and High Targeted Expression (HTE) platforms. a**) High Targeted Expression (HTE) platforms express the capsid library under the same spleen-focus-forming virus (SFFV) promoter construct containing the SV40 intron as used to drive eGFP expression in the Functional Transduction (FT) platform [Figure 1b]. The capsid library coding region is followed by internal ribosomal entry sites (IRES) from either the human eIF4G1 gene (eHTE) or the encephalomyocarditis virus (EMCV; vHTE) enabling downstream eGFP expression. The selection procedure is identical to library selection using the FT platform resulting in alternative methods e/vHTE-DNA (capsids recovered by amplifying capsid DNA that entered target cells) and e/vHTE-RNA (capsids recovered by amplifying capsid mRNA that was expressed in target cells). **b**) Plasmids required for AAV production using the Replication Competent (RC), FT, and HTE platforms. The RC platform contains Rep2 and the desired *cap* gene flanked by AAV2-ITRs and, therefore, only adenoviral helper genes are required *in trans* to enable AAV particle formation. The FT and HTE platforms do not contain most of the Rep2 gene and require an additional Rep2∆Cap plasmid construct *in trans* for productive packaging. **c**) Total vector yields as total vector genomes (vg) per single 15 cm plate productions for RC, FT, vHTE, and eHTE using AAV2, AAV8 and AAV-DJ capsids (n=9) P-values: 0.05 > * ≥ 0.01 > ** ≥ 0.001 > *** according to non-parametric t-test (Mann-Whitney) compared to the respective RC condition. **d-f**) Production of AAV2, AAV8, and AAV-DJ in the FT (**d**), vHTE (**e**), and eHTE (**f**) platforms with two different Rep2 helper plasmids, wild-type and one containing ATG to ACG mutation to decrease Rep78 and Rep68 expression. **g**) FT platforms with GFP expression driven by the ubiquitous spleen focus-forming virus (SFFV) promoter, the liver-specific promoter (LSP), the neuron-specific human synapsin 1 (hSYN) promoter and the muscle-specific myosin light chain (MLC) promoter. **h**) AAV-DJ produced with the different FT constructs shown in [**g**].

**
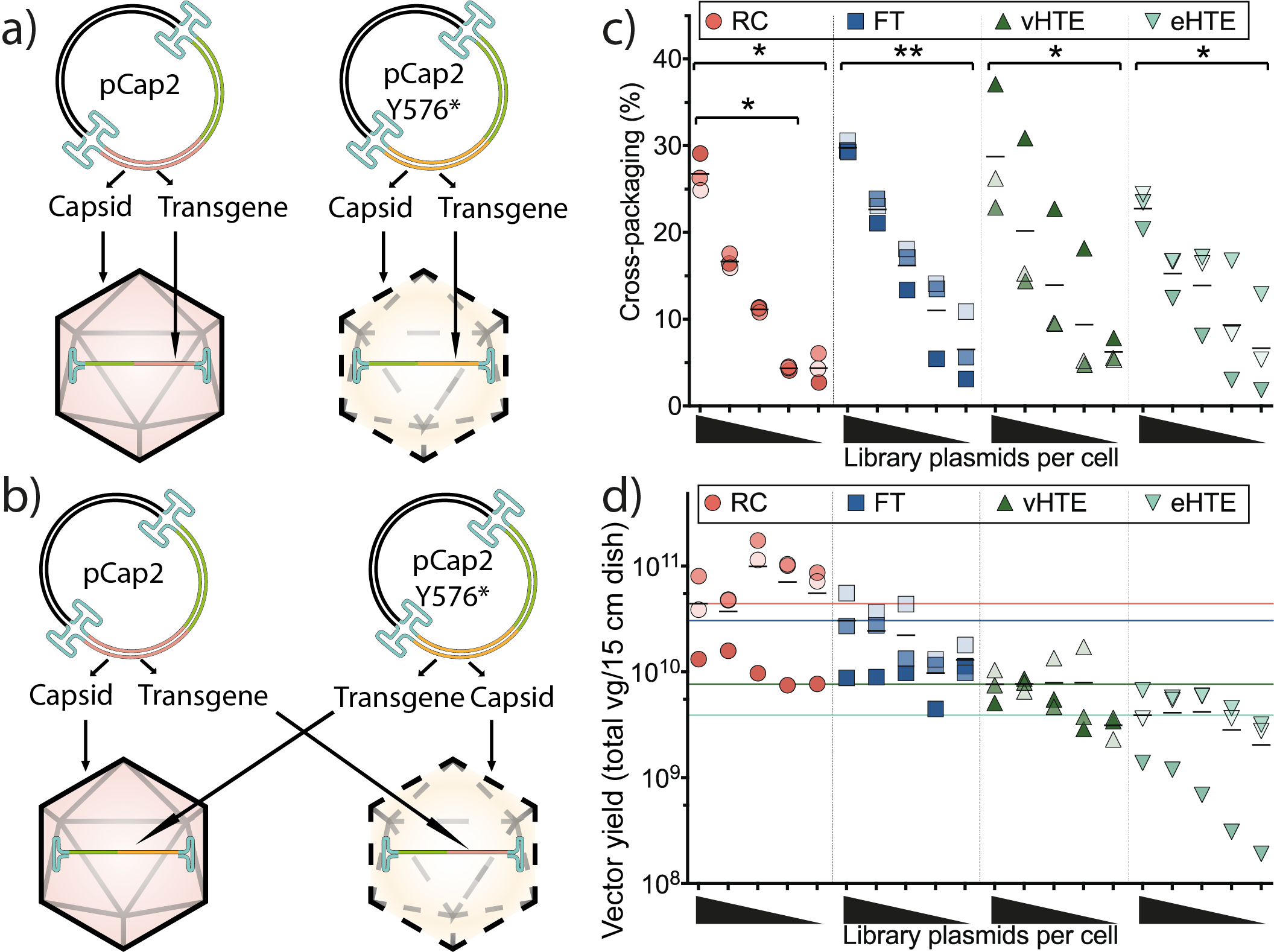
**

**Supplementary Figure 2. Analysis of cross-packaging in the Replication Competent (RC), Functional Transduction (FT) and High Targeted Expression (HTE) platforms. a-b**) Schematic showing the experimental approach for cross-packaging analysis. Correct packaging was defined as wild-type *cap2* sequence contained in DNaseI-resistant particles [**a**], while cross-packaging was defined as detection of the cap2 Y576* sequence from DNaseI-resistant particles [**b**]. Detection was performed using Illumina amplicon-seq. **c**) Proportion of cross-packaging measured by NGS detection of AAV Cap2 with a stop codon (Y576*). wtCap2 and Cap2-Y576* plasmids were mixed at 1:1 ratio using different total amounts of library plasmid. n=3; plasmids per cell (from left to right, as indicated by ticks): 25,000, 10,000, 5,000, 1,000, 500. (p-values, Kruskal-Wallis test with Dunn’s comparison to the 25,000 condition: 0.05 > * ≥ 0.01 > **). Data points from the same set of experiments are coded in the same shade of the indicated color. **d**) Total vector yields as total vector genomes (vg) per single 15 cm plate productions for RC, FT, vHTE, and eHTE platforms transfected with a 1:1 mix of Cap2 and Cap2 Y576* in single 15 cm plate productions (n=3) at various amounts of library plasmids per cell, keeping Rep2∆Cap and pAd5 plasmids constant. Mean titers of the respective 25,000 plasmids/cell condition are indicated with horizontal black lines for RC (red circle), FT (blue square), vHTE (green triangle) and eHTE (teal reversed triangle) (plasmids per cell: 25,000, 10,000, 5,000, 1,000, 500). Data points from the same set of experiments are coded in the same shade of color.


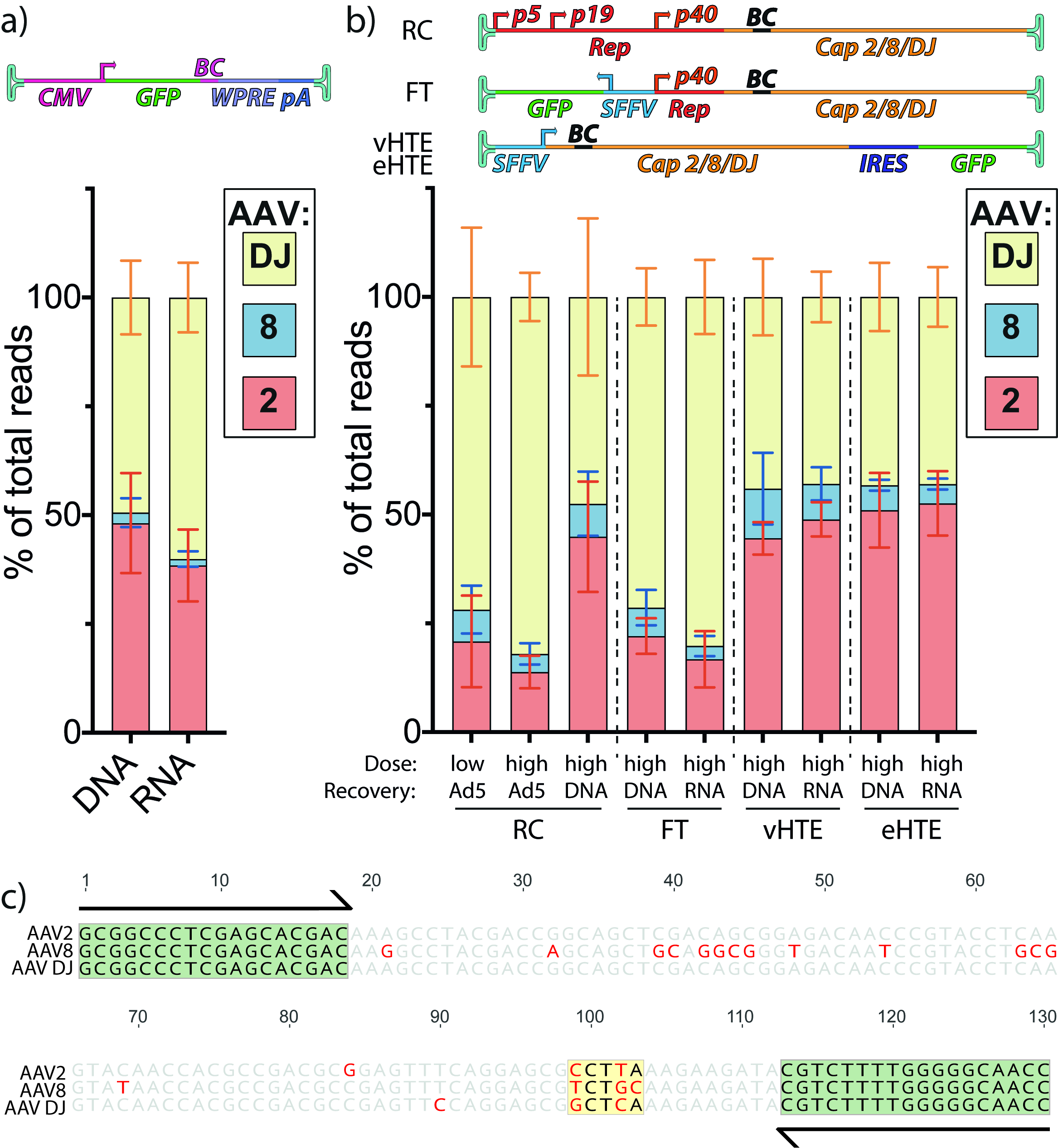


**Supplementary Figure 3. Comparison of the Replication Competent (RC), Functional Transduction (FT) and High Targeted Expression (HTE) platforms based on stringency of the selection process. a**) AAV2, AAV8, and AAV-DJ capsid performance on HuH-7 cells based on DNA and RNA NGS data using a barcoded (BC) CMV-GFP transgene. **b**) Individually packaged AAV2, AAV8, and AAV-DJ were produced using the RC, FT, vHTE, and eHTE (n=7) platforms, mixed at 1:1:1 (AAV2:AAV8:AAV-DJ) ratios and used to transduce HuH-7 cells (n=7) for a single round of selection each and processed according to the indicated recovery methods. The RC platform was used at two doses in the context of Ad5 super-infection (50 [low] and 500 [high] AAV vg/cell). All other conditions were transduced at a combined dose of 500 [high] AAV vg/cell. **c**) Schematic of largely common regions of capsid genes from AAV2, AAV8 and AAV-DJ, utilized for internal NGS analysis in [**b**]. Consensus nucleotides are shown in black font, differences from the consensus and non-consensual positions shown in red font. The common PCR primer binding sites for amplicon NGS are displayed with a green background; the ‘naturally-occurring’ barcode-index used to differentiate the capsid gene contribution are displayed with a yellow background.

**
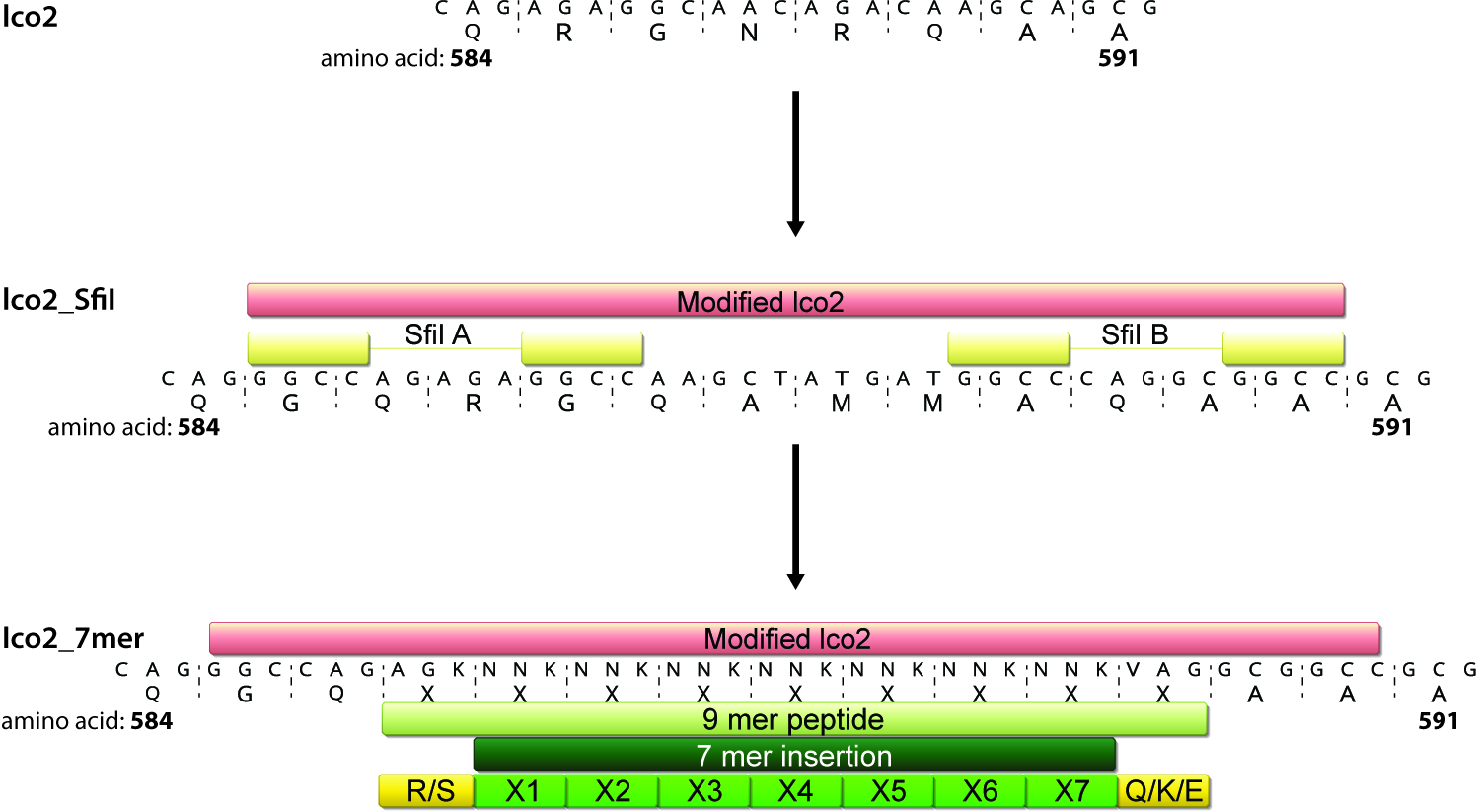
**

**Supplementary Figure 4. Peptide library construction.** Detailed view of the modification workflow of the lco2 capsid. Amino acid position of Q584 and A591 using numbering from un-modified cap2 VP1. The first step was the insertion of two SfiI restriction sites. The second step was the insertion of the peptide library. Seven truly randomized NNK (X1-X7) insertions as well as the full 9mer peptide including semi-random flanking amino acids are shown within the modified region. Abbreviations: lco: local codon-optimized.^22^

**
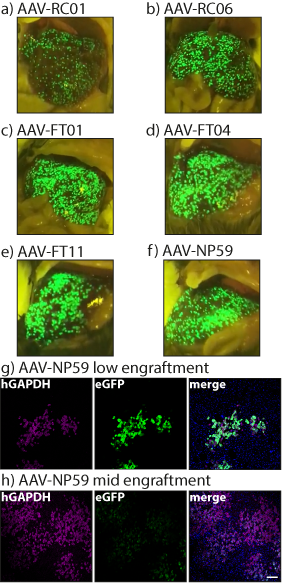
**

**Supplementary Figure 5. Supplementary information for AAV2 peptide display variant validation. a-f**) Macroscopic images of native GFP expression of AAV-injected humanized FRG mice. **g&h**) Representative immunofluorescence images of human hepatocytes transduced with AAV NP59. Human hepatocytes were engrafted at low [**g**] and medium [**h**] levels into FRG mice and labeled with anti-human glyceraldehyde-3-phosphate dehydrogenase (hGAPDH). The scale bar represents 100 µm and is valid for images in [**g**] and [**h**]. **h**) For convenience of direct comparison, images from **Fig. 3g** are shown here as panel (**h**).

**
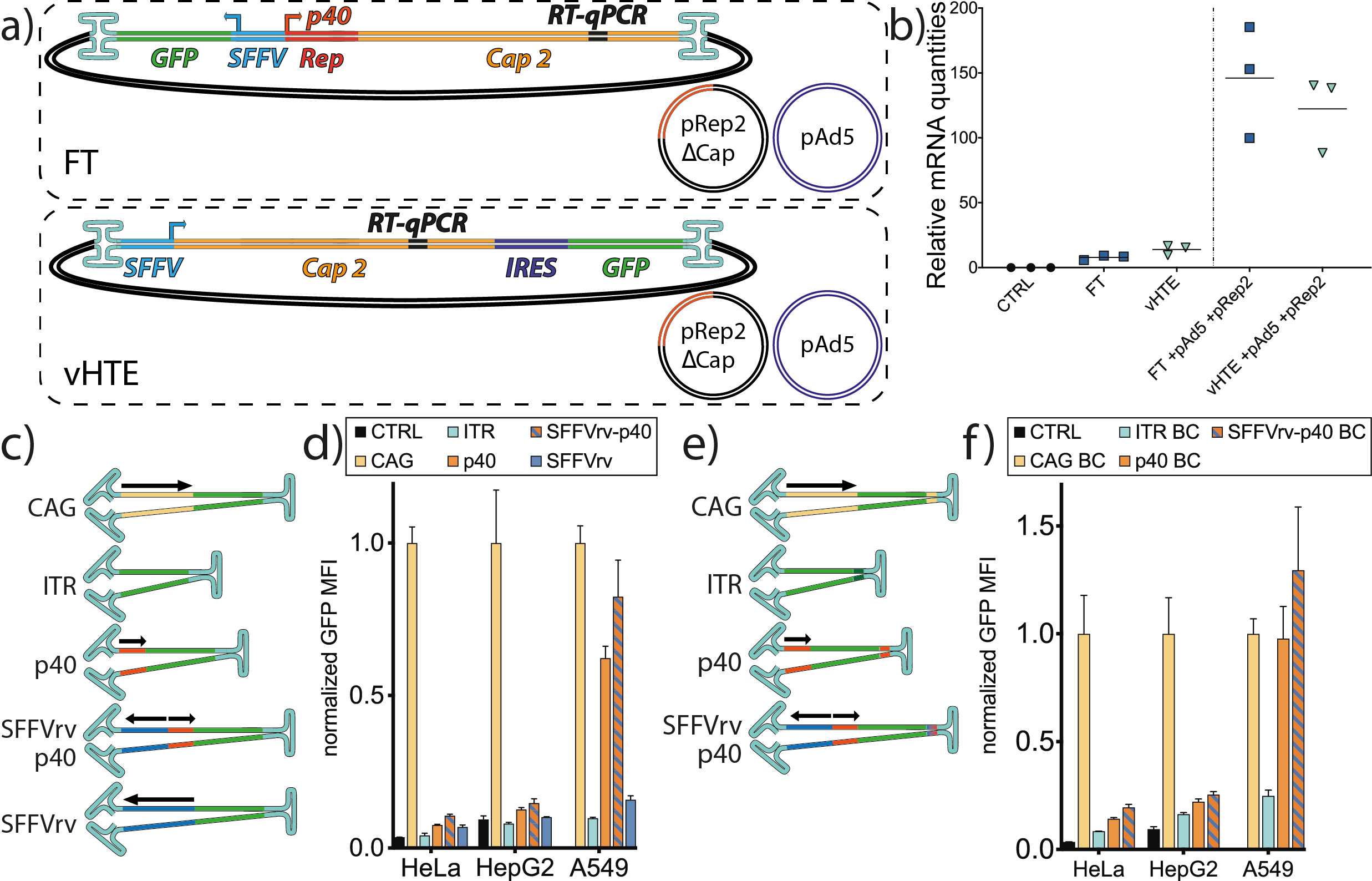
**

**Supplementary Figure 6. Rep2-p40-driven RNA expression *in vitro*. a**) FT (upper) and vHTE (lower) constructs used in plasmid transfection experiment with the RT-qPCR amplifications region indicated. **b**) Transfection of HEK293T cells. Relative mRNA quantities (RQ = 2^-(Ct[Cap2]-Ct[βactin])^) of Cap2 compared to beta-actin in absence and presence of helper plasmids. **c**) scAAV promoter constructs packaged into AAV-7m8 and used in [**d**]. **d**) Mean fluorescence intensity (MFI) of cells transduced with non-barcoded scAAV-’Promoter’-GFP constructs shown in [**c**]. All MFIs were normalized to MFI of scAAV-CAG-GFP, which was assigned value =1. Multiplicity of transduction (MOT)=1,000 vector genomes(vg)/cell, N=3 for HeLa, HepG2, and A549. **e**) Barcoded scAAV-7m8-packaged promoter constructs. **f**) MFI of barcoded scAAV-’Promoter’-GFP constructs from [**e**]. GFP MFI was normalized to MFI of scAAV-CAG-GFP. MOT=1000 vg/cell, N=3 for HeLa, HepG2 and A549. Abbreviations: pAd5: helper plasmid with adenoviral helper functions; FT: functional transduction platform; vHTE: viral high targeted expression platform; CAG: Cytomegalovirus enhancer - chicken β-actin promoter - globin intron; p40: Rep2-p40 promoter; ITR: inverted terminal repeat; SFFV: spleen focus-forming virus promoter.


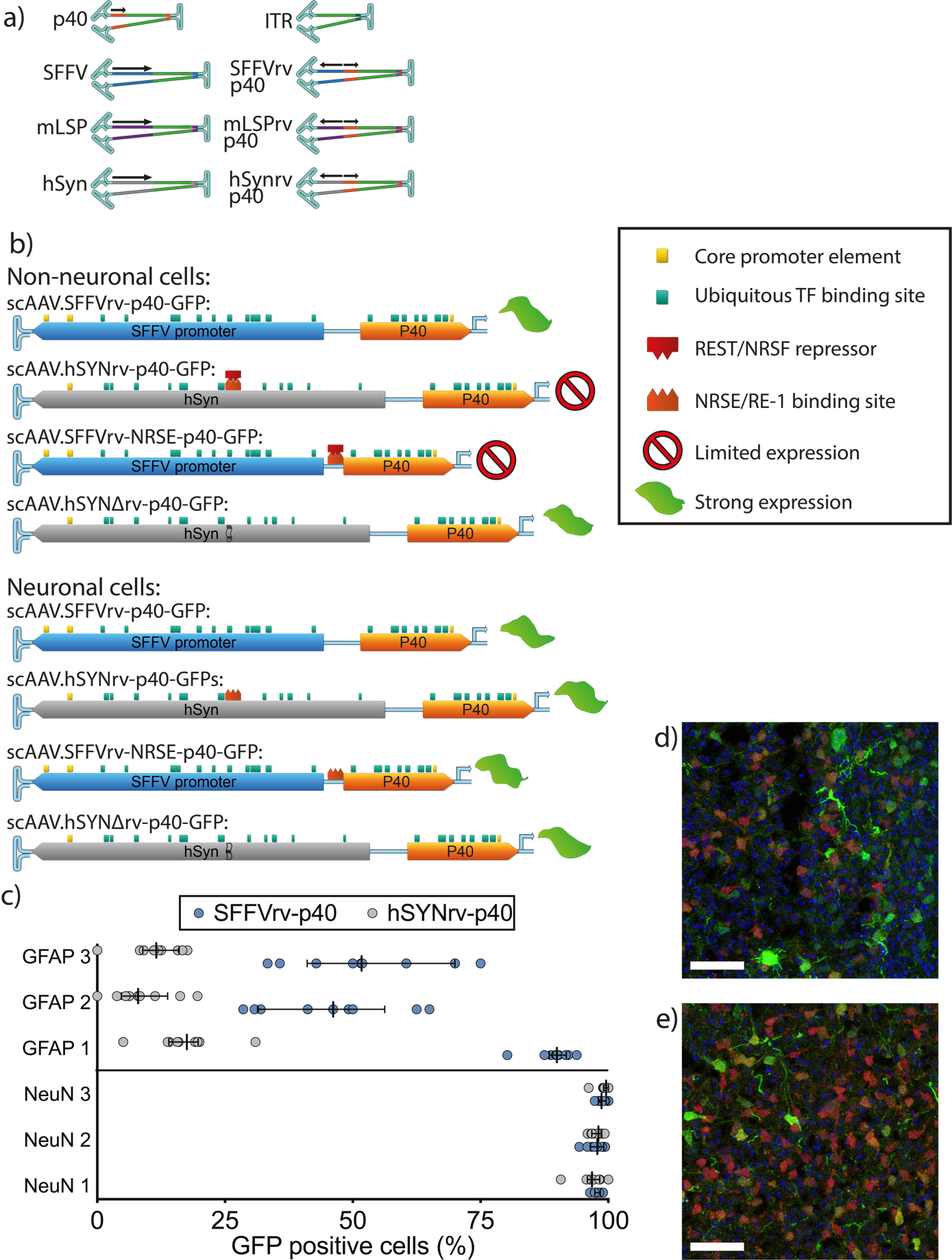


**Supplementary Figure 7. AAV-p40-hybrid promoter constructs used to modify gene expression. a**) Uniquely barcoded scAAV9-packaged promoter constructs used in [**Fig. 5a**]. **b**) Proposed model of neuron-specific p40-activity by incorporation of the NRSE/RE-1 binding site into the p40-hybrid promoter constructs. Data from evaluation of displayed constructs are shown in **Fig. 5b-f**. **c**) Individual data from evaluation of transduced organoids (see **Fig.** **5d-f**). each circle represents the percentage of GFP-positive cells per image taken. Data was collected individually for cortical organoids of different origin. **d-e**) Induced pluripotent stem cell (iPSC)-derived cortical organoid transduced with AAV-7m8.SFFVrv-p40-GFP [**d**] or AAV-7m8.hSYNrv-p40-GFP [**e**]. All cells were stained with DAPI (blue), neuronal nuclear antigen (NeuN)-positive neurons were stained for NeuN expression (red), and transduced cells were stained green using anti-GFP antibody. Scale bar represents 50 µm. Abbreviations: p40: Rep2-p40 promoter; ITR: inverted terminal repeat; SFFV: spleen focus-forming virus promoter; mLSP: minimal liver-specific promoter; hSyn: human synapsin 1 promoter; TF: transcription factor; REST: Repressive element 1 silencing transcription factor; NRSF: Neuron-restrictive silencing factor; NRSE: neuron restrictive silencing element; RE-1: repressive element 1; GFAP: glial fibrillary acidic protein; NeuN: neuronal nuclear antigen

**Supplementary Table 1. Highest ranking peptides in all steps of the selection process**

| **Allocated variant name** | **Replication competent** | **Unique peptides** | **Percent of original** |
| --- | --- | --- | --- |
|  | **Packaged library** | **633363** | **100 %** |
|  | **Peptide** | **Read counts** | **Percent of total reads** |
|  | SIRPPITRQ | 1657 | 0.03 % |
|  | RGNRQPESK | 1283 | 0.02 % |
|  | RLFNQAPQQ | 1165 | 0.02 % |
|  | RGNSVTTHK | 948 | 0.02 % |
|  | RRQPVSRQE | 870 | 0.02 % |
|  | SRPPFVAAK | 581 | 0.01 % |
|  | SLYSSPQKQ | 445 | 0.01 % |
|  | SSMLPPRPQ | 392 | 0.01 % |
|  | RPNGGQTLK | 359 | 0.01 % |
|  | SVTRTKGPQ | 353 | 0.01 % |
|  | SPHMSTPTK | 344 | 0.01 % |
|  | SRLTPRADQ | 341 | 0.01 % |
| **Allocated variant name** | **Replication competent** | **Unique peptides** | **Percent of original** |
|  | **Round 1 – Ad5** | **47196** | **7.45 %** |
|  | **Peptide** | **Read counts** | **Percent of total reads** |
|  | STELNGQLE | 1211 | 0.04 % |
|  | SRREGSVLK | 1143 | 0.03 % |
|  | STSSRSAPQ | 1029 | 0.03 % |
|  | RNVGEPREE | 998 | 0.03 % |
|  | SKGRVVTPE | 844 | 0.03 % |
|  | RGAGSGGWE | 766 | 0.02 % |
|  | SEQTKSREQ | 725 | 0.02 % |
|  | SSPSPEPNQ | 710 | 0.02 % |
|  | SGRSKGDSE | 694 | 0.02 % |
|  | RSVEVREDE | 668 | 0.02 % |
|  | RGELGGRPE | 659 | 0.02 % |
|  | RQTAEKTSK | 646 | 0.02 % |
| **Allocated variant name** | **Replication competent** | **Unique peptides** | **Percent of original** |
|  | **Round 2 – Ad5** | **10214** | **1.61 %** |
|  | **Peptide** | **Read counts** | **Percent of total reads** |
| **(hu.Hep.)RC01** | STTHLSPPQ | 4398505 | 75.88 % |
| **(hu.Hep.)RC02** | SELEEMNNK | 194604 | 3.36 % |
| **(hu.Hep.)RC03** | SPSSPAPAQ | 121492 | 2.10 % |
| **(hu.Hep.)RC04** | RPETQAKPQ | 113161 | 1.95 % |
| **(hu.Hep.)RC05** | STAYTPAPQ | 98450 | 1.70 % |
| **(hu.Hep.)RC06** | RSQRETVWK | 76763 | 1.32 % |
| **(hu.Hep.)RC07** | SVMMVGGRE | 69334 | 1.20 % |
| **(hu.Hep.)RC08** | RKDPEVSEQ | 59689 | 1.03 % |
|  | STTLAHTLE | 38604 | 0.67 % |
|  | RQALENPRE | 36843 | 0.64 % |
|  | SSTPSPPQK | 34753 | 0.60 % |
|  | RGVLDGLEQ | 29158 | 0.50 % |
| **Allocated variant name** | **Functional Transduction** | **Unique peptides** | **Percent of original** |
|  | **Packaged library** | **4192002** | **100 %** |
|  | **Peptide** | **Read counts** | **Percent of total reads** |
|  | SPPPPPPPQ | 3903 | 0.04 % |
|  | RPPPPPPPQ | 1003 | 0.01 % |
|  | SPTPPPPPQ | 956 | 0.01 % |
|  | SPPPPTPPQ | 827 | 0.01 % |
|  | SPPTPPPPQ | 820 | 0.01 % |
|  | SPPPPPTPQ | 789 | 0.01 % |
|  | SPPPRPPPQ | 780 | 0.01 % |
|  | SPPSPPPPQ | 769 | 0.01 % |
|  | SPPPTPPPQ | 762 | 0.01 % |
|  | SPSPPPPPQ | 725 | 0.01 % |
|  | SPPPPPPTQ | 714 | 0.01 % |
|  | SPPPPSPPQ | 712 | 0.01 % |
| **Allocated variant name** | **Functional Transduction** | **Unique peptides** | **Percent of original** |
|  | **Round 1 – DNA** | **1445870** | **34.49 %** |
|  | **Peptide** | **Read counts** | **Percent of total reads** |
| ***(hu.Hep.)RC05*** | STAYTPAPQ | 962 | 0.03 % |
|  | SPPPPPPPQ | 543 | 0.02 % |
|  | SPPRPPPPQ | 332 | 0.01 % |
|  | SPPPRPPPQ | 328 | 0.01 % |
|  | RPPPPPPPQ | 281 | 0.01 % |
|  | SPPPPRPPQ | 267 | 0.01 % |
|  | SPTPPPPPQ | 235 | 0.01 % |
|  | SPRPPPPPQ | 226 | 0.01 % |
|  | SPPPAPPPQ | 225 | 0.01 % |
|  | SPPAPPPPQ | 215 | 0.01 % |
|  | SPPTPPPPQ | 195 | 0.01 % |
|  | SPHPPPPPQ | 191 | 0.01 % |
| **Allocated variant name** | **Functional Transduction** | **Unique peptides** | **Percent of original** |
|  | **Round 1 – RNA** | **13623** | **0.32 %** |
|  | **Peptide** | **Read counts** | **Percent of total reads** |
|  | RDDSAPHQR | 29434 | 0.86 % |
|  | RPAPPPSPE | 25157 | 0.73 % |
|  | RSPPPPSSQ | 20351 | 0.59 % |
|  | SKWTRPTRK | 20227 | 0.59 % |
|  | SHLSAPPAK | 19743 | 0.58 % |
|  | SLPLPPPTQ | 19392 | 0.57 % |
|  | STATRPAFQ | 18876 | 0.55 % |
|  | SPPPPSYTQ | 18542 | 0.54 % |
|  | RSPSKFPQK | 18434 | 0.54 % |
|  | SPTPPPPPQ | 18312 | 0.53 % |
|  | RQPQRPPPE | 17944 | 0.52 % |
|  | RML*PGPLE | 17943 | 0.52 % |
| **Allocated variant name** | **Functional Transduction** | **Unique peptides** | **Percent of original** |
|  | **Round 2 – RNA** | **5510** | **0.13 %** |
|  | **Peptide** | **Read counts** | **Percent of total reads** |
| **(hu.Hep.)FT01** | SKTNLDRAQ | 166047 | 6.49 % |
| **(hu.Hep.)FT02** | SATQPYASQ | 148539 | 5.80 % |
| **(hu.Hep.)FT03** | RTSAHQVGE | 104192 | 4.07 % |
| **(hu.Hep.)FT04** | RSPKGSAWE | 89065 | 3.48 % |
| **(hu.Hep.)FT05** | SPQQPPRSQ | 89000 | 3.48 % |
| **(hu.Hep.)FT06** | SSRTPQHKQ | 82167 | 3.21 % |
| **(hu.Hep.)FT07** | SPRHPVTTQ | 81175 | 3.17 % |
| **(hu.Hep.)FT08** | SHTTGSPAK | 68639 | 2.68 % |
| **(hu.Hep.)FT09** | RMTGKTGYE | 67346 | 2.63 % |
| **(hu.Hep.)FT10** | RQATSGFDK | 66337 | 2.59 % |
| **(hu.Hep.)FT11** | RAKEMRSEQ | 66318 | 2.59 % |
| **(hu.Hep.)FT12** | SAFSQSAVK | 65274 | 2.55 % |

**Supplementary Table 2. FRG mouse human albumin levels**

| **Experiment** | **Human albumin levels (mg/mL)** | **Estimated Replacement Index (RI, %)** |
| --- | --- | --- |
| RC-2pep Round 1 | 2.16 | 20 - 30 |
| RC-2pep Round 2 | 2.53 | 20 - 30 |
| FT-2pep Round 1 | 1.00 | _~_10 |
| FT-2pep Round 2 | 3.35 | 30 - 40 |
| Candidate validation 1 | 3.10 | 30 - 40 |
| Candidate validation 2 | 2.38 | 20 - 30 |
| Candidate validation 3 | 1.34 | 10 - 20 |
| Candidate validation 4 | 1.21 | 10 - 20 |
| IHC RC01 | 0.77 | < 10 |
| IHC RC06 | 1.16 | 10 - 20 |
| IHC FT01 | 2.54 | 20 - 30 |
| IHC FT04 | 2.10 | 20 - 30 |
| IHC FT11 | 4.43 | 40 - 50 |
| IHC NP59 low engraftment | 0.73 | < 10 |
| IHC NP59 mid engraftment | 4.39 | 40 - 50 |
| NGS high engraftment 1 | 12.69 | > 90 |
| NGS high engraftment 2 | 13.38 | > 90 |

**Supplementary Table 3. Primers used in the presented study**

| **Name** | **Sequence (5’->3’)** | |
| --- | --- | --- |
| **Cloning** | **Sequence (5’->3’)** | |
| SFFV_MluI_F | GACGCGTGTAACGCCATTTTGCAAGGCATGG | |
| SFFV_NotI_R | GGCGGCCGCCGACTCAGTCTGTCGGAGGACTG | |
| hSyn_MluI_F | GCACGCGTGTGTCTAGACTGC | |
| hSyn_NotI_R | TATTGCGGCCGCCTTCTCGACTGCGCTCTC | |
| MLC_MluI_F | TATTACGCGTCGACCCAGAGCACAGAGC | |
| MLC_NotI_R | TATTGCGGCCGCTGGCCGGCCCCTG | |
| eIRES_NsiI_F | TTCCATATGCATAGATGGGGGTCCTGGG | |
| eIRES_MfeI_R | GGTGGCCAATTGCCTCCTTGGTTTGGATCTCG | |
| mut_pA_1 | CGAATTAAACGGTCGCTTGATTAACAAGCAATTACAG | |
| mut_pA_2 | CTTGTTAATCAAGCGACCGTTTAATTCGTTTCAGTTG | |
| p40_AvrII_F | CCGCAGTTTCCCTAGGAAGGTCACCAAGCAGGAAG | |
| p40_BamHI_R | GCAGCTGGATCCGACTCGCGCACCCG | |
| SFFVrv_AvrII_F | ACCTTAGAGCCCTAGGCGACTCAGTCTGTCGGAGG | |
| lco2_SfiI_1 | GGCCATCATAGCTTGGCCTCTCTGGCCCTGGAGGTTGGTAGACACAG | |
| lco2_SfiI_2 | GGCCAAGCTATGATGGCCCAGGCGGCCGCGACCGCAGATGTCAAC | |
| lco2_NNK7 | ATCTGCGGTCGCGGCCGCCTBMNNMNNMNNMNNMNNMNNMNNMCTCTGGCCCTGGAGGTTGGT | |
| lco2-dsSyn | ACCAACCTCCAGGGCCAGAG | |
| PepRec_F | GACGAAGAGGAAATTAGGACCACTAAC | |
| PepRec_R | CTGCAGGTACACGTCTCTG | |
| **qPCR/ddPCR** | **Sequence (5’->3’)** | |
| eGFP_F | TCAAGATCCGCCACAACATC | |
| eGFP_R | TTCTCGTTGGGGTCTTTGCT | |
| Rep2_F | AAGGATCACGTGGTTGAGGT | |
| Rep2_R | CCCACGTGACGAGAACATTT | |
| b-actin_F | CTCGCCTTTGCCGATCC | |
| b-actin_R | GGGGTACTTCAGGGTGAGGA | |
| wtCap2_F | AGACGAAGAGGAAATCAGGACAACC | |
| wtCap2_R | CCATGCCTGGAAGAACGCC | |
| **cDNA synthesis** | **Sequence (5’->3’)** | |
| wtAAV8/lco2_R | CGGTTTATTGATTAACAGGCAATTACAGATTACG | |
| wtAAV2/DJ_R | CGGTTTATTGATTAACAAGCAATTACAGATTACG | |
| WPRE_R | GGATTTATACAAGGAGGAGAAAATGAAAG | |
| **Next-generation sequencing** | **Primer barcode (5’->3’)** | **Main oligo sequence (5’->3’)** |
| Cross-Pack_F | n/a | TTTGGGAAGCAAGGCTCAGAG |
| Cross-Pack_R01 | AGCC | GCTTGTCTGTTGCCTCTCTGG |
| Cross-Pack_R02 | TACA | GCTTGTCTGTTGCCTCTCTGG |
| Cross-Pack_R03 | TGCG | GCTTGTCTGTTGCCTCTCTGG |
| Cross-Pack_R04 | CTTC | GCTTGTCTGTTGCCTCTCTGG |
| Cross-Pack_R05 | TGAC | GCTTGTCTGTTGCCTCTCTGG |
| Cross-Pack_R06 | GAGC | GCTTGTCTGTTGCCTCTCTGG |
| Cross-Pack_R07 | ATCA | GCTTGTCTGTTGCCTCTCTGG |
| Cross-Pack_R08 | GATG | GCTTGTCTGTTGCCTCTCTGG |
| Cross-Pack_R09 | AGAG | GCTTGTCTGTTGCCTCTCTGG |
| Cross-Pack_R10 | TCGA | GCTTGTCTGTTGCCTCTCTGG |
| 2-8-DJ_F | n/a | GCGGCCCTCGAGCACGAC |
| 2-8-DJ_R1 | AGCC | GGTTGCCCCCAAAAGACG |
| 2-8-DJ_R2 | TACT | GGTTGCCCCCAAAAGACG |
| 2-8-DJ_R3 | TGCG | GGTTGCCCCCAAAAGACG |
| 2-8-DJ_R4 | CTTC | GGTTGCCCCCAAAAGACG |
| 2-8-DJ_R5 | TGAC | GGTTGCCCCCAAAAGACG |
| 2-8-DJ_R6 | GAGC | GGTTGCCCCCAAAAGACG |
| PepLib_F | n/a | CTAACCCTGTGGCCACGG |
| PepLib_R | n/a | CGTCTCTGTCTTGCCACACC |
| GFP_BC_WPRE_F01 | GTTCA | GCTGGAGTTCGTGACCGCCG |
| GFP_BC_WPRE_F02 | GTCAT | GCTGGAGTTCGTGACCGCCG |
| GFP_BC_WPRE_F03 | CTGTA | GCTGGAGTTCGTGACCGCCG |
| GFP_BC_WPRE_F04 | GTATT | GCTGGAGTTCGTGACCGCCG |
| GFP_BC_WPRE_F05 | CTAGT | GCTGGAGTTCGTGACCGCCG |
| GFP_BC_WPRE_F06 | ACTTC | GCTGGAGTTCGTGACCGCCG |
| GFP_BC_WPRE_F07 | CCTAT | GCTGGAGTTCGTGACCGCCG |
| GFP_BC_WPRE_F08 | ACTGA | GCTGGAGTTCGTGACCGCCG |
| GFP_BC_WPRE_F09 | TCCAA | GCTGGAGTTCGTGACCGCCG |
| GFP_BC_WPRE_F10 | GCATT | GCTGGAGTTCGTGACCGCCG |
| GFP_BC_WPRE_F11 | TCAAG | GCTGGAGTTCGTGACCGCCG |
| GFP_BC_WPRE_F12 | TCAGA | GCTGGAGTTCGTGACCGCCG |
| GFP_BC_WPRE_F13 | TCGTA | GCTGGAGTTCGTGACCGCCG |
| GFP_BC_WPRE_F14 | ACGAT | GCTGGAGTTCGTGACCGCCG |
| GFP_BC_WPRE_F15 | TATCC | GCTGGAGTTCGTGACCGCCG |
| GFP_BC_WPRE_F16 | CATGA | GCTGGAGTTCGTGACCGCCG |
| GFP_BC_WPRE_F17 | CACTC | GCTGGAGTTCGTGACCGCCG |
| GFP_BC_WPRE_F18 | GACTA | GCTGGAGTTCGTGACCGCCG |
| GFP_BC_WPRE_F19 | TACCC | GCTGGAGTTCGTGACCGCCG |
| GFP_BC_WPRE_F20 | GACAT | GCTGGAGTTCGTGACCGCCG |
| GFP_BC_WPRE_F21 | GAAGA | GCTGGAGTTCGTGACCGCCG |
| GFP_BC_WPRE_R | n/a | CAACATAGTTAAGAATACCAGTCAATCTTTCAC |
| SV40pA_R | n/a | ATTGCAGCTTATAATGGTTACAAATAAAGC |
| **Novel capsid cloning** | **Sequence (5’->3’)** | |
| RC01_F | AGTCCTCCGCAGGCGGCCGCGACCGCAG | |
| RC01_R | CAGATGAGTAGTACTCTGGCCCTGGAGGTTGG | |
| RC02_F | GATGAATAATAAGGCGGCCGCGACCGCAG | |
| RC02_R | TCCTCAAGCTCACTCTGGCCCTGGAGGTTGG | |
| RC03_F | TGCTCCTGCGCAGGCGGCCGCGACCGCA | |
| RC03_R | GGCGAAGAAGGACTCTGGCCCTGGAGGTTGG | |
| RC04_F | GGCGAAGCCGCAGGCGGCCGCGACCGCAG | |
| RC04_R | TGCGTCTCCGGCCTCTGGCCCTGGAGGTTGG | |
| RC05_F | TCCTGCGCCGCAGGCGGCCGCGACCGCA | |
| RC05_R | GTATAAGCAGTACTCTGGCCCTGGAGGTTGG | |
| RC06_F | GACGGTGTGGAAGGCGGCCGCGACCGCA | |
| RC06_R | TCCCTCTGAGACCTCTGGCCCTGGAGGTTGG | |
| RC07_F | GGGGGGTCGTGAGGCGGCCGCGACCGCA | |
| RC07_R | ACCATCATAACACTCTGGCCCTGGAGGTTGG | |
| RC08_F | GGTTTCTGAGCAGGCGGCCGCGACCGCA | |
| RC08_R | TCAGGATCCTTCCTCTGGCCCTGGAGGTTGG | |
| FT01_F | AGTAAGACGAATCTTGATCGGGCGCAGGCGGCCGCGACCGCAGATG | |
| FT02_F | AGTGCGACTCAGCCTTATGCGAGTCAGGCGGCCGCGACCGCAGATG | |
| FT03_F | AGGACTTCTGCGCATCAGGTGGGGGAGGCGGCCGCGACCGCAGATG | |
| FT04_F | AGGTCGCCGAAGGGTTCGGCGTGGGAGGCGGCCGCGACCGCAGATG | |
| FT05_F | AGTCCTCAGCAGCCGCCTCGTTCTCAGGCGGCCGCGACCGCAGATG | |
| FT06_F | AGTTCGCGGACTCCGCAGCATAAGCAGGCGGCCGCGACCGCAGATG | |
| FT07_F | AGTCCGCGTCATCCTGTGACGACTCAGGCGGCCGCGACCGCAGATG | |
| FT08_F | AGTCATACGACGGGTAGTCCTGCTAAGGCGGCCGCGACCGCAGATG | |
| FT09_F | AGGATGACTGGGAAGACTGGTTATGAGGCGGCCGCGACCGCAGATG | |
| FT10_F | AGGCAGGCTACGAGTGGGTTTGATAAGGCGGCCGCGACCGCAGATG | |
| FT12_F | AGTGCTTTTTCGCAGTCGGCGGTTAAGGCGGCCGCGACCGCAGATG | |
| Universal_FT_R | CTGGCCCTGGAGGTTGGTAGACACAG | |
| FT11_F | GCGGTCGGAGCAGGCGGCCGCGACCGCAGATGTCAAC | |
| FT11_R | ATCTCCTTAGCCCTCTGGCCCTGGAGGTTGGTAGACACAG | |
